# New ecosystems in the deep subsurface follow the flow of water driven by geological activity

**DOI:** 10.1038/s41598-019-39699-w

**Published:** 2019-03-01

**Authors:** G. Borgonie, C. Magnabosco, A. García-Moyano, B. Linage-Alvarez, A. O. Ojo, L. B. Freese, C. Van Jaarsveld, C. Van Rooyen, O. Kuloyo, E. D. Cason, J. Vermeulen, C. Pienaar, E. Van Heerden, B. Sherwood Lollar, T. C. Onstott, S. O. C. Mundle

**Affiliations:** 1 Extreme Life Isyensya (ELI), PB 65, 9050 Gentbrugge, Belgium; 2 Flatiron Institute Center for Computational Biology, Simons Foundation, 162 5th Ave., New York, 10010 USA; 3 Centre for Applied Biotechnology, Uni Research AS Thormøhlensgate 55, N-5008 Bergen, Norway; 4 Department of Microbial, Biochemical and Food Biotechnology, Swot Street, 9300 Bloemfontein, Republic of South Africa; 5 AngloGold Ashanti Kopanang Mine, Private Bag X5010, Vaal Reef, North West, 2621 Republic of South Africa; 6 Northam Platinum Ltd., Zondereinde Division, Farm Zondereinde 384KQ, District Thabazimbi, Limpopo Province, Republic of South Africa; 7 Department of Geoscience, University of Calgary, 2500 University Drive Northwest Calgary, Alberta, Canada T2N 1N4; 8 Biosaense,Walter Sisulu 5, Bloemfontein, 9031 South Africa; 9 Department of Earth Sciences, University of Toronto, 22 Russell Street, Toronto, Ontario Canada M5S 3B1; 10 Department of Geosciences, Princeton University, B79 Guyot Hall, Princeton, 08544 New Jersey USA; 11 Great Lakes Institute for Environmental Research, University of Windsor, 401 Sunset Ave., Windsor, ON Canada N9B 3P4; 12 NORCE Norwegian Research Centre, Marine Biotechnology - University of Bergen, Department of Biological Sciences, Thormøhlens gate 53/55, 5008 Bergen, Norway

## Abstract

Eukarya have been discovered in the deep subsurface at several locations in South Africa, but how organisms reach the subsurface remains unknown. We studied river-subsurface fissure water systems and identified Eukarya from a river that are genetically identical for 18S rDNA. To further confirm that these are identical species one metazoan species recovered from the overlying river interbred successfully with specimen recovered from an underlying mine at −1.4 km. *In situ* seismic simulation experiments were carried out and show seismic activity to be a major force increasing the hydraulic conductivity in faults allowing organisms to create ecosystems in the deep subsurface. As seismic activity is a non-selective force we recovered specimen of algae and Insecta that defy any obvious other explanation at a depth of −3.4 km. Our results show there is a steady flow of surface organisms to the deep subsurface where some survive and adapt and others perish. As seismic activity is also present on other planets and moons in our solar system the mechanism elucidated here may be relevant for future search and selection of landing sites in planetary exploration.

## Introduction

Eukarya have been identified in deep subsurface fracture waters of South Africa^1–4^ but the mechanisms that transport organisms to the subsurface are not well understood^5,6^. Previous studies suggested that the majority of the Eukarya identified in the subsurface were genetically more closely related to surface freshwater sources, rather than soil^4^. This is supported by the general lack of Eukarya in soils beyond a few meters from the surface and suggests that terrestrial pathways are unlikely^7^.

**Figure 1:**
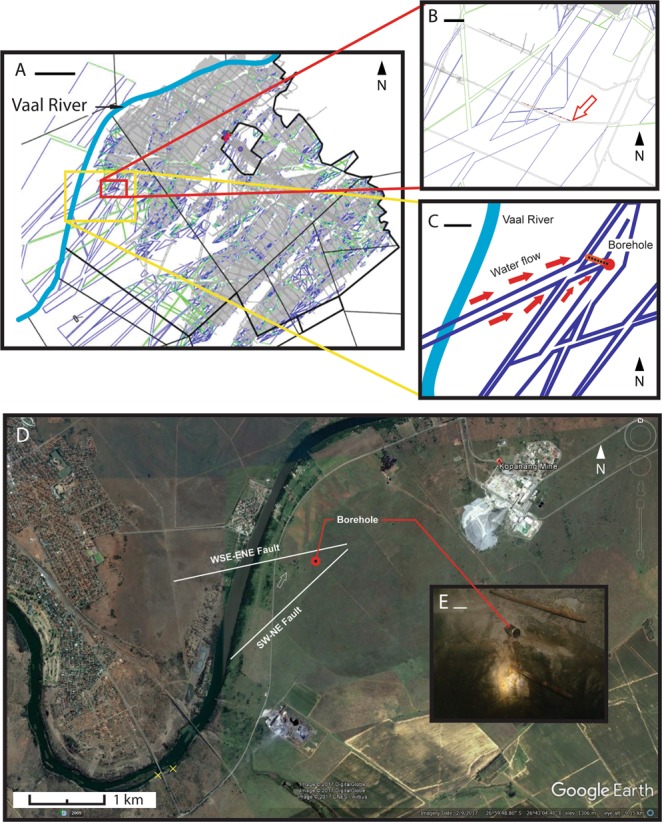
Kopanang-Vaal system. (**A**) Map of Kopanang Mine showing dykes (green lines) fault zones (blue lines) and mining tunnels, stopes (grey), Vaal River (light blue) and property boundaries (black lines). Red box is inset outline for B. Yellow box is inset outline for C. (**B**) A simplified geological map of the mine level showing the location of the borehole (red arrow) with respect to two intersecting fault zones (outlined in blue), one striking WSW/ENE and a younger fault zone striking SW/NE and off-setting the old fault zone, showing the dykes (green), mining tunnels (grey lines), and the borehole (brown line with blue dots). The open red arrow marks position of the drilling cubby where the borehole is accessible from the tunnel. (**C**) The possible direction of fissure water flow along the fault zone and towards the borehole (red arrows). Also shown is the position of the Vaal River overhead (light blue) hypothesized to recharge the fissure fluid. (**D**) Google Earth image of the region showing the location of Kopanang Mine and the vertical extrapolation of the location of the borehole (red dot) and the fault zones. Sampling sites of Vaal River water and mud occur near Orkney bridge (yellow X’s). The open white arrow points to an isolated patch of vegetation sitting on top of a ridge with the WSW/ENE fault zone to the north and the SW/NE fault zone to the south. Red dot marks position of the drilling cubby where the borehole is accessible from the tunnel. (**E**) Photograph of the borehole in the cubby. Scale bars: (**A**) 1 km, (**B**) 100 m, (**C**) 250 m, (**D**) 1 km, (**E**) 10 cm. Map data: Google, Digitalglobe/CNES/Airbus.

The Republic of South Africa hosts the world’s deepest mines, some of which exceed 4 km below land surface (kmbls). The network of tunnels and crosscuts in these gold (Au), platinum (Pt) and diamond mines allow exceptional access to the deep subsurface. During the course of normal mining operations, the advancing tunnels and exploratory boreholes facilitate sampling by intersecting water-bearing fractures. These boreholes typically occur in small stations on one side of the tunnel. The migration pathways for surface waters carrying organisms and nutrients into the deep subsurface can be impacted by a number of geological parameters. Identification of deep life ecosystems across a range of subsurface conditions and temporal scales suggests the driving forces are likely non-specific (e.g. thermal and seismic) and occur on geological timescales. Evidence for seismic activity in South Africa over geological timescales is supported by African historical epicentral locations, where there are in the wide plate boundary zone (up to 1600 km wide) belt-like zones of seismicity surrounding relative aseismic blocks^8^. The seismic belts are mainly coincident with rifts but not all of these rifted regions experience strong earthquakes. Also seismicity belts do occur where no rift faults are apparent. In the Republic of South Africa a belt of seismic activity extends N-S along the RSA-Mozambique border (Lembobo Mountains), southwards into KwaZulu-Natal and another trend E-W through southern KwaZulu-Natal, Lesotho and the southern Free State. The Cape Fold Belt and the adjacent Karoo Basin are equally subject to sporadic activity. We refer to the study of Brandt (2011)^8^ particularly his Fig. 1 where all seismicity between 1620 and 2010 is mapped showing that South Africa has natural seismic activity although by world standards very moderate and of a shallow character. Occasional bursts of seismic activity have occurred at numerous places in South Africa (we refer also to the database reported in Midzi (2013)^9^.

Historical data suggests continuous natural tectonic activity occurred in South Africa over geological time suggesting that seismic forces may be a contributing factor for the development of deep subsurface ecosystems. However, it is generally accepted that mining causes anthropogenic seismic activity^10,11^. Anthropogenic seismicity began when earthquakes occurred in Johannesburg in 1894; by 1908 these events had been attributed to the Witwatersrand gold production, which had commenced in 1886^10^. Today, especially around the area of Klerksdorp, Welkom and Carletonville, earthquakes are registered (40/month) that are the result of mining activity^11^. Higher fissure water flow and seismic activity above baseline levels will accelerate and reinforce existing hydrogeological processes.

The genetic link of organisms in deep subsurface ecosystems to freshwater sources implies that migration occurs along specific pathways from surface point sources. The timing of migration is constrained by the local hydrogeology, where more open systems are impacted by meteoric recharge, and more closed systems have been isolated over longer timescales. Less isolated hydrogeologic systems provide a unique opportunity to evaluate the mechanisms that transport and support life in the deep subsurface, provided that enough geological, chemical and biological information exists to link a deep mine borehole to an overlying freshwater source.

## Results

Sampling sites were chosen at two different mines representing different geographic locations, geologies and depths. Study locations were selected with overlying surface water bodies, previously known deep ecosystems and open hydrological systems.

To determine the validity of a direct link between surface and deep subsurface, we analyzed the geological setting, aqueous chemistry, and the biological species content of the surface water and fissure water. We investigated the relationship between surface freshwater and deep fracture water at two locations, Kopanang gold mine (−1.4 km) and Zondereinde platinum mine (−1.7 km), situated near South Africa’s Vaal and Bierspruit Rivers, respectively. The indigenous Eukarya recovered from the fracture water at both locations were collected using previous stringent methods^1–4^(Supplementary information). Fracture water samples were additionally analyzed for chemistry and microbiological content and compared with five sediment samples from the Bierspruit River and eight samples from the Vaal River. Additionally we performed and videotaped *in situ* experiments to study the dynamic behavior of biofilm inside fissures under different stresses acting on the fracture water flow.

### Geological and aqueous chemistry link between surface and borehole

**Figure 2:**
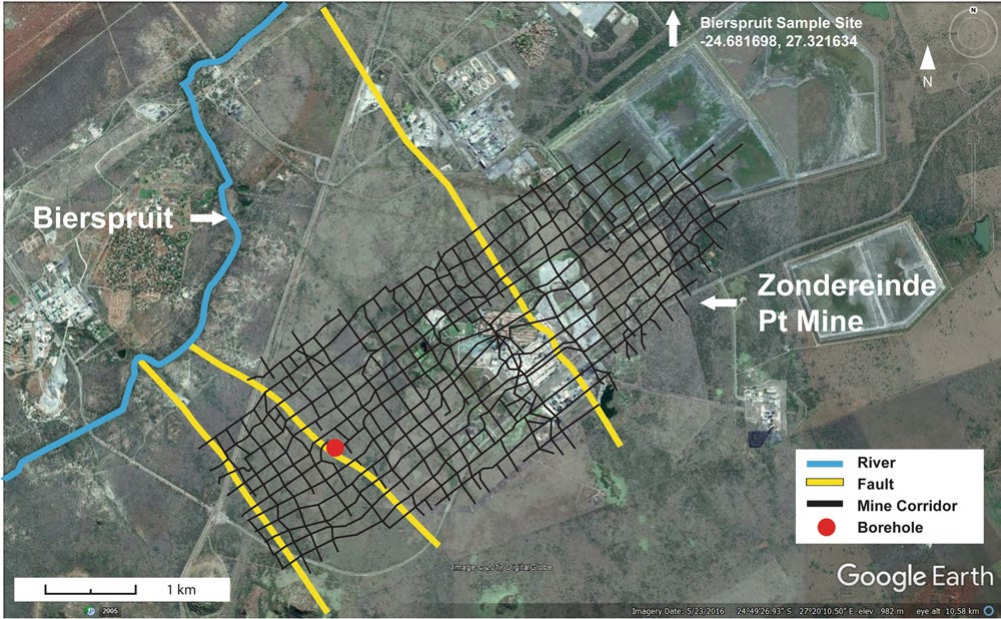
Zondereinde-Bierspruit system. Google Earth image of Western Bushveld Igneous Complex mining region and mining map of Zondereinde juxtaposed upon it. Black lines demarcate mine property, stopes and tunnels. Dykes strike SE-NW and faults (yellow lines) strike N-S and ESE-WSW. The borehole (red dot) lies directly on the fault leading to the Bierspruit River. Scale bar 1 km. Map data: Google, Digitalglobe.

**Figure 3:**
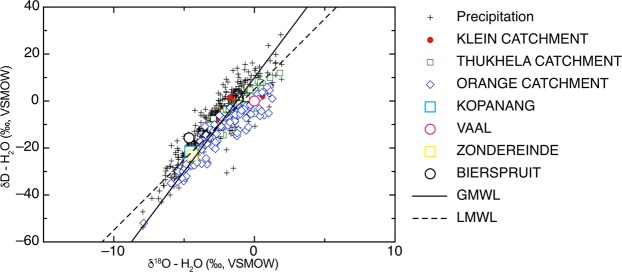
δ^18^O and δD values for Kopanang Mine (blue square, this study), Zondereinde Mine (yellow square, this study), Vaal River (pink circle, this study), and Bierspruit River (black circle, this study). Solid line represent the global meteoric water line (GMWL, δD = 8(^18^O) + 10), dashed line represents estimated mean rain line for Southern Africa (MRWL, δD = 6(^18^O) + 5) (I.A.E.A, 1981), red circles represents river water values from the Klein Catchment, blue diamonds represents river water values from the Orange Catchment, green squares represents river water values from the Thukhela Catchment, and black ‘ + ’ symbols represent values for location precipitation (Data from GNIR and GNIP databases).

A network of faults was identified linking the borehole in the Kopanang Mine to the Vaal River (Fig. 1) and the Zondereinde-Bierspruit geological map also shows a fault linking the borehole to the Bierspruit River (Fig. 2). The age and origin of fracture fluids (modern vs. paleometeoric) in South Africa have been well characterized using a number of approaches^12^. The δ^18^O-H_2_O and δD-H_2_O values are consistent with groundwater supply wells in the area^13^. The ^14^C-DIC (<1 to 2768 years) and ^3^H (7–10 years) results show (Table 1, Fig. 3) that the fracture water recovered from Kopanang mine was impacted by recent meteoric recharge. ^14^C-DIC (5798 years) supported recent reports that the Zondereinde mine fracture water was influenced by paleometeoric water (Table 1, Fig. 3)^13^

**Table 1:** Physico-chemical and biological characteristics of the fissure water in Kopanang-Vaal and Zondereinde-Bierspruit. (mg/L unless otherwise stated), CFU: colony forming unit. BDL: below detection limit. ND: not determined, NA: not applicable. Control samples is treated mine water used for cooling equipment and drinking water.

	Kopanang	Vaal	Zondereinde	Bierspruit		
Depth	−1.4	NA	−1.7	NA		
Flow rate (L/min.)	3.15	NA	8	NA		
Sample size (L)	3,865,654	15	8640	4.5		
^14^C age	<1 to 2768^(4)^	NA	5798^(1)^	NA		
^3^H age	7–10 yr^(4)^	NA	NA	NA		
Microbial cells/litre	<10^3^	1.4 × 10^7^	2 × 10^5^	2.2x10^6^		
**Planktonic**						
T(°C)	31.3	18.6	45.3	20.8		
pH	8.15	8.37	9.3	8.34		
EC (mS/m)	47.9	58.1	130	156		
TDS (ppm)	370	397	650	966.46		
O_2_d	0.1(M)	23(µM)	0.3(M)	24(µM)		
TOC	2.54	3.41	8.8	19.78		
DOC	0.9	3.92	3.6	7.49		
Al^3+^	0.054	0.090	0.25	<0.004		
Ba^2+^	0.040	0.027	0.04	0.020		
Ca^2+^	47.9	58.1	13.3	39.91		
Cd^2+^	bdl	bdl	bdl	bdl		
Cl^−^	16.5	53.4	76.1	26.9		
Cr^2+^	<0.006	<0.006	0.005	0.006		
Cu^+^	0.006	0.008	0.003	0.005		
Fe_total_	0.005	0.023	0.0234	0.017		
Mg^2+^	0.76	18.80	3.6	69.83		
Mn^2+^	0.015	0.015	0.27	0.007		
Mo^2+^	<0.003	<0.003	0.005	<0.003		
Na^+^	111.3	48.83	228	173.02		
Ni^2+^	<0.010	<0.010	<0.010	<0.010		
K^+^	0.74	7.83	7.14	2.92		
Si^4+^	8.717	1.061	10.2	2.796		
Sr^2+^	0.046	0.085	0.9	0.044		
V^3+^	<0.006	<0.006	<0.006	<0.006		
Zn^2+^	0.023	0.014	0.018	0.016		
F^−^	0.25	0.22	0.38	0.78		
NO_2_^−^	bdl	bdl	0.083	bdl		
Br^−^	0.0468	0.0914	2.38	1.08		
NO_3_^−^	0.0482	1.675	0.937	0.59		
PO_4_^3−^	bdl	bdl	0.033	0.035		
SO4^2−^	29.7	97	29.1	99		
NH_4_^+^	0.12	0.19	0.15	0.251		
*E.coli* (cfu/100ml)	0	>20	ND	>9.0		
Faecal coliforms (cfu/100ml)	0	>1000	ND	>1000		
***Biodiversity***						
***Phylum***	***shared genetic identity (%)***					
	***Kopanang***	***Vaal***	***18S***	***cross***	***Zondereinde Bierspruit***	
**PROTOZOA**						
*Cyclidium*	+	+	100%	NA	−	−
**PLATHELMINTHES**						
*Dochmiotrema*	+	+	100%	NA	−	−
*Stenostomum* sp.	+	−	−	−	−	−
**NEMATODA**						
*Tridentulus* sp.	−	+	−	−	−	+
*Tobrilus gracilis*	−	−	−	−	−	+
*Poikilolaimus oxycercus*	+	+	100%	F6	−	−
*Halicephalobus gingivalis*	+	−	−	−	−	−
**ANNELIDA**						
*Aeolosoma hemprichi*	+	−	−	−	−	−
**ROTIFERA**						
*Rotifera sp*.	+	−	−	−	−	−
Control Samples	−	−	−	−	−	−

### Biological content

Molecular analyses revealed identical 18S rRNA gene sequences between protozoan, Nematoda and Platyhelminthes specimens of subsurface borehole water (Kopanang mine) and the surface Vaal River water (Table 1). No Eukarya were recovered from the Zondereinde borehole (Table 1); therefore, a biological comparison was not possible. The failure to physically or molecularly recover any Eukarya from Zondereinde may be due to the increased residence times of the fluids. The microbial composition studies on samples of the Vaal River (STable 1) show no overlap with the Kopanang borehole microbial community in this, or previous studies^4,14^.

### Nematode morphometrics and crossing experiments

Based on the species concept in biology the strongest evidence, besides morphometrics (Table 2) and genetic analysis, that species from the surface and subsurface are the same is to check for viable offspring when crossed. The nematode *Poikilolaimus oxycercus* was the only species collected on the surface and subsurface having male/female specimen where crossing tests could be completed.

**Table 2:** Morphometrics of the nematode species *Poikilolaimus oxycercus* collected from the Kopanang gold mine borehole and the nearby Vaal River. Morphometrics does not show significant differences. In classical nematode taxonomy based on these data both specimens would be considered one and the same species. ***Poikilolaimus oxycerca*** n= 5 females a: body length/maximal width; b: body length/oesophagus length; c:body length/tail length; V: distance of vulva from anterior as percentage of total length.

	Kopanang	Vaal River
L (micrometer)	1203 ± 91.2 (1100–1324)	1060 ± 116.1 (903–1213)
Maximum width (micrometer)	76 ± 8.2 (65–85)	74.8 ± 17.6 (49–84)
Oesophagus length (micrometer)	189.4 ± 6.6 (179–195)	170.8 ± 6.8 (160–178)
Tail length (micrometer)	45 ± 1.6 (43–47)	43 ± 1.9 (41–46)
a	15.9 ± 1.4 (14.2–17.5)	15.5 ± 1.9 (13.2–18.4)
b	6.4 ± 0.7 (5.6–7.4)	6.1 ± 0.7 (5.4–7.1)
c	27.6 ± 2.1 (25.6–30.7)	24.6 ± 2.8 (21–28.8)
V	56.2 ± 1.5 (54–58)	56.4 ± 1.1 (55–58)

**Figure 4:**
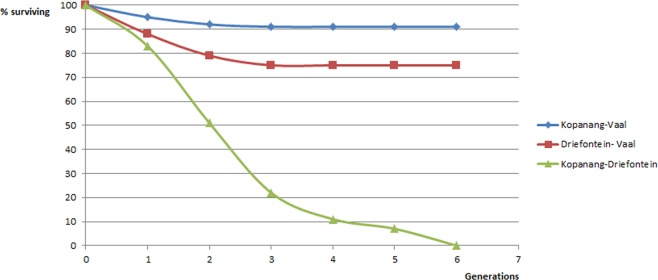
Percentage of surviving lines versus generations in crossing experiments between *Poikilolaimus oxycercus* species for six generations. The cross between the Vaal Rivers specimen and the Kopanang Mine and Driefontein Mine specimens were healthy; whereas the cross between the Kopanang Mine and Driefontein Mine specimens floundered by the sixth generation.

Morphometric analysis of *P. oxycercus* species from the Vaal river and from Kopanang mine confirmed that the Nematoda recovered from these localities were most likely identical species (Table 2). To determine convincingly if *P. oxycercus* collected from the borehole at Kopanang^4^ is the same species as the specimen collected from the Vaal river, surface- and subsurface-isolated *P. oxycercus* were crossed to determine if they produce viable offspring. Viable offspring for *P. oxycercus* were only recovered from Kopanang gold mine and Vaal River crosses (Fig. 4). Additional crosses were done with *P. oxycercus* recovered from another deep mine, Driefontein gold mine^4^, 114 km away. Figure 4 shows that the Kopanang-Vaal cross and Driefontein-Vaal yield viable and healthy offspring. However the Kopanang-Driefontein cross does not last beyond F6.

### *In situ* experiments

**Figure 5:**
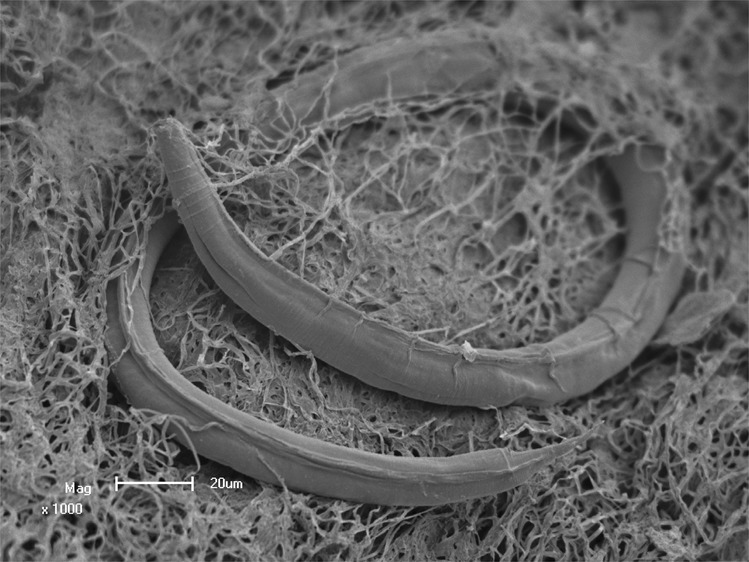
Scanning electron Microscopy of a small biofilm piece captured during seismic *in situ* experiment. In Kopanang Mine 138 pieces of biofilm were captured in the period 20–40 minutes after the borehole was reopened. Six pieces contained nematodes. All exhibited the same curled up appearance as in this picture indicating the specimens were in their survival stage (dauer stage) in suspended animation. Four of the six pieces were transferred to a petri dish and reawakened and were subsequently identified as *Poikilolaimus oxycercus*. Scale bar: 20 µm.

To test fluid transport and biological migration into the subsurface, three *in situ* experiments were performed. First, video cameras were installed inside the boreholes and mechanically simulated a seismic event by pulsing the flow of water (pressure build up and release, SVideo 1). This experiment demonstrated that the sudden change in flow released particles in the borehole (SVideo 2). The particles were captured in the outflow and identified with SEM as pieces of dislodged biofilm; although no Eukarya were observed. We also observed that the sudden change in flow released dissolved gases from the fracture water which contributed to dislodging biofilm (SVideo 3). In the second experiment, we characterized the biofilm from a flowing borehole, closed the borehole for 6 weeks, then subsequently re-established flow for 6 weeks and re-evaluated the biofilm. The creation of an artificial cul de sac killed the biofilm within 6 weeks; however, the biofilm grew back within 6 weeks when the flow was re-established (SVideo 4–6). In the third experiment completed in Kopanang mine, a flowing borehole was closed for 5 months and, upon re-establishment of the flow, the fracture water was collected, filtered, chemically analyzed and Heterotrophic Plate Count (HPC) tested on 20 min intervals over a period of 80 minutes (Table 3). The results demonstrated that HPC and biofilm pieces peaked in the initial 20–40 min of outflow from the borehole. Six out of 138 biofilm pieces contained nematodes in survival stage (Fig. 5), four nematodes became active upon transferal to a Petri dish, and identified as *P. oxycercus* that were previously recovered in Kopanang fracture water^4^.

**Table 3:** Seismic mimic experiment at Kopanang. After closure of the borehole for 30 min. the flow was released suddenly and sampled for chemical composition and HPC every 20 min. for 80 minutes. BDL: below detection limit. mg/L unless otherwise stated. Number of biofilm pieces reported are those collected in the 20 minute time frame.

	+20 min	+40 min	+60 min	+80 min
Volume(L)	101	80	72	63
T(°C)	31.3	31.3	32.0	32.4
pH	8.15	8.10	8.17	8.16
EC (mS/m)	47.9	49.1	49.1	49.1
TDS (ppm)	370	378	374	366
O_2_d	23	bdl	bdl	bdl
TOC	2.54	1.88	1.23	1.93
DOC	0.9	0.7	0.5	0.7
Al^3+^	0.054	0.041	0.029	0.039
Ba^2+^	0.040	0.023	0.032	0.028
Ca^2+^	47.9	49.1	49.1	49.1
Cd^2+^	<0.001	<0.001	<0.001	<0.001
CL^−^	16.5	15.9	15.7	15.6
Cr^2+^	<0.006	<0.006	<0.006	<0.006
Cu^3+^	0.006	0.004	0.003	0.006
Fe	0.005	0.004	0.000	0.012
Li	0.011	0.015	0.014	0.013
Mg^2+^	0.76	0.82	0.74	0.74
Mn^2+^	0.015	0.007	0.010	0.010
Mo^2+^	<0.003	<0.003	<0.003	<0.003
Na^+^	111.3	120.35	116.95	110.91
Ni^2+^	<0.010	<0.010	<0.010	<0.010
K^+^	0.74	1.11	0.84	0.61
Pb	<0.010	<0.010	<0.010	<0.010
Si^4+^	8.717	8.555	8.616	8.707
Sr^2+^	0.046	0.024	0.033	0.037
V^3+^	<0.006	<0.006	<0.006	<0.006
Zn^2+^	0.023	0.014	0.012	0.011
F	0.25	0.24	0.23	0.24
Cl	16.5	15.9	15.7	15.6
NO_2−_	bdl	bdl	bdl	bdl
Br^−^	0.0468	0.0639	0.061	0.086
NO_3−_	0.0482	−0.05	−0.05	−0.05
PO_4_^3−^	bdl	bdl	bdl	bdl
SO_4_^2−^	29.7	30.045	29.865	29.288
NH_4_^+^	0.12	0.18	0.06	0.17
Tot hard	9.96	11.24	10.04	9.80
HPC	6	>1000	0	0
# biofilm pieces	30	138	25	32

### Rate of descent into the subsurface

In order to evaluate how quickly eukaryotes may be transported to the subsurface, triangulation was used to calculate the speed of the fracture water flow from the Vaal river to Kopanang gold mine and the Bierspruit river to Northam Platinum mine. This yields an average distance and speed that does not account for differences in flow speed across flow paths caused by geology, porosity and inclination. Triangulation provides a reasonable estimate based on the linearity of the facture and limited information available related to the geological setting along the entire length of the fracture. An additional limitation with this approximation is the assumption that the Vaal-Kopanang and Bierspruit-Zondereinde systems were isolated from other fracture water input. Most fracture waters are mixed from various sources; however, more detailed information was not available.

The distance between the Vaal river and the geographic coordinates of the Kopanang borehole is 3.9 km with the borehole at −1.4 km. These distances yield an estimated fracture water travel distance of 4.14 km and an ^3^H-derived water age of 7–10 years^4^ (Table 1). The distance between the Bierspruit river and the geographic coordinates of the Zondereinde borehole is 8 km with the borehole at −1.7 km, yielding a fracture water travel distance of 8.18 km and ^14^C-derived water age of 5798 years^1^ (Table 1). Using this method, we estimate that fracture water travels at a migration rate of 591 m yr^−1^ to 414 m yr^−1^ in the Kopanang-Vaal system and at a migration rate of 0.7 m yr^−1^ in the Zondereinde-Bierspruit system.

### Unexpected Eukarya recovered from the deep subsurface

If seismic activity enables Eukarya to reach the deep subsurface it implies that, since this force is non-selective, organisms would be recovered from fracture water that would be unable to survive from a biological point of view. Since 2006 samples were taken from more than 50 deep subsurface boreholes and yielded from three different mines in five independent samples; three Insecta (two Coleoptera; a scutellum piece at Star diamond mine at −640 m, *Hydroglyphus pusillus (Coleoptera)* at Evander mine at −1.7 km and *Anisochrysa* possibly *A. carnea* (Hymenoptera) at Driefontein mine at −1.0 km).Two samples with algae (Chlorophyta (green alga), were recovered from two mines. *Chlorella* and *Mesotaenium* at Tau Tona at −3.4 km. *Chlorella, Mesotaenium* and *Crucigenia* at Star Diamond at −640 m (SVideo 7, SFig. 2). Except one sample at Evander, all samples were taken under stringent aseptic conditions and exclude contamination from mine activities as a source. Life cycle, dietary needs, morphology and physiological limitations suggest that these specimens should not be able to survive in the deep subsurface.

### Metatranscriptomic analyses

To independently assess whether or not algae and Insecta were present in the subsurface fluids, metatranscriptomic analyses were carried out on fracture water collected from 3 different mines (Beatrix (BE326) 2011, 2012, Tau Tona (TT109) and Finsch (FI88) STable 2). Mapping of the metatranscriptomic reads to a 16S rRNA gene database identified RNA sequences related to several eukaryote taxa within the deep mines (STable 4). Taxa that were represented by more than one RNA sequence in any metatranscriptome were filamentous (Basidiomycota) fungi, sac (Ascomycota) fungi, algae, diatoms, ciliates, Amoebozoa, and flatworms (STable 2).

Although 16S rRNA gene transcripts are a good indicator for which taxa are present and active in the subsurface community, they do not provide insight into the interactions between eukaryotes and prokaryotes in these environments. If we are to regard the eukaryotes identified in the subsurface as lingering life forms that are nearing (or have reached) death due to starvation, an interaction we might observe is an active bacterial community breaking down these nutrient-rich eukarya. To test this hypothesis, we performed targeted assemblies of microbial chitinase, pectinase, and cellulase protein encoding genes (PEGs) which would, theoretically, be capable of degrading eukaryal cells. If translated, these enzymes may be used by bacteria and/or archaea to degrade eukaryal cell components. Cellulase-related transcripts were identified in Beatrix and Tau Tona fracture water but chitinase and pectinase were not.

## Discussion

The geological maps confirmed a direct link between surface river and subsurface fracture water. When combined with the chemical compositions (Table 1) of the fracture water, the δ^18^O-H_2_O and δD-H_2_O values and the ^14^C-DIC and ^3^H results suggest that more open hydrogeologic systems can exhibit water penetrations to extraordinary depth, with short subsurface residence times that are consistent with migration from the overlying river water sources (Table 1). Only the biological content of the Vaal river-Kopanang borehole could be compared and based on DNA analysis revealed similar species at both sample locations. This is supported by the successful cross made between the nematode *P. oxycercus* from the Vaal river and *P. oxycercus* from the Kopanang borehole. The observation that the Kopanang-Driefontein cross of *P. oxycercus* is not viable beyond F6 was observed before^4^ and we hypothesize that this is due to the earlier isolation event from the surface which represents an already severe inbreeding step for both species. Alternatively, it may indicate a speciation process is underway either in the Kopanang recovered species or in the Driefontein species.

The geological, chemical and biological data make a convincing case for biological transport within the Kopanang-Vaal system and that the Vaal river is the origin of the protozoan, Nematoda and Platyhelminthes specimens recovered in the deep subsurface. Unfortunately, a biological comparison could not be done in the Zondereinde-Bierspruit system but the geology and water chemistry also support a direct link.

Based on triangulation the estimate is that fracture water travels at a migration rate of 591 m yr^−1^ to 414 m yr^−1^ in the Kopanang-Vaal system and at a migration rate of 0.7 m yr^−1^ in the Zondereinde-Bierspruit system. Accounting for the influences of an open borehole, the latter approximation is close to the theoretical travel speeds of 3 × 10^–3^ to 0.3 m yr^−1^ proposed for terrestrial cratons^5^ whereas the former is consistent with the accelerated migration rates associated with dewatering processes in mines^15^. Notably, the estimated rates in this calculation will be higher than anticipated under natural conditions from dewatering in the mines.

Seismic activity is known to induce significant hydrological changes in the subsurface. Seismic-induced changes to fracture apertures are known to increase hydraulic conductivity and decrease water table levels^16–18^. We propose that seismic activity is a major driving force for the movement of nutrients and organisms through structural faults. Splitting/expanding fractures/faults in the subsurface creates conduits that facilitate migration of microbes and Eukarya and support their survival by providing access to new food sources. Sudden changes in fracture water flow from seismic activity could dislodge and transport biofilm to new (pristine) rock faces. Seismic activity can also have a negative impact on deep ecosystems by decreasing rock permeability and water flow to create dead-ends for existing biofilm and planktonic communities. The selective sweep that was suggested to have led to the “single-species” community of “*Ca. D. audaxviator*” at −2.8 km in South Africa may be one such example^19^.

The three *in situ* experiments suggest that sudden changes in pressure and fracture water flow rates can stimulate the displacement of biofilms and their inhabitants in the subsurface, where seismic activity is the natural analogue of these experiments. We deliberately selected mine-river systems that were impacted by more recent recharge waters as they were more likely to demonstrate recent migration of organisms from surface water sources into the fracture waters of deep mines. However, seismic events that occurred between 1620 and 2010 (visualized in Brandt, 2011^8^) demonstrate that seismic activity is a natural occurrence in South Africa that predates mining. Based on the fact that we previously recovered Eukarya in older fracture waters from more isolated hydrogeologic systems with ages that considerably predate mining activity^1–4^, our current model provides the most likely mechanism for the descent and survival of organisms in the deep biosphere. The time-dependence and causality associated with the development of deep ecosystem in different hydraulic systems suggests that the timing of the creation of water migration pathways by seismic activity need not coincide with the movement of organisms to the subsurface. For example, ancient networks of fractures and porosity can be generated by seismic activity that can be exploited by subsequent climate changes leading to water flow at later geological timescales. Conversely, areas with ancient water flow pathways to the deep subsurface can become isolated by seismic activity (ancient open hydrogeological systems that are closed systems).

Seismic activity can change hydrogeological systems and structures that impact water migration pathways that support deep ecosystems. However, water flow can also be impacted by other structural and thermal driving forces^20^. Similar to seismic activity, thermal hotspots can cause fracturing and drive hydrological systems on longer timescales than seismic events. Seismic activity can help provide pathways but is not needed to drive the flow. These thermal forces may not have the same scale of impact on dislodging biofilms in the deep subsurface; however, they can provide passive mechanisms for the movement of organisms and nutrients over geological timescales supporting life between the large changes triggered by seismic events. Permeability of different stratigraphic zones can also impact deep ecosystems. Permeable regions will provide conduits to different areas of the subsurface and potentially provide larger volume water reservoirs at different depths in the subsurface that sustain life as it migrates to deeper depths. The development and support of deep ecosystems is dependent on the flow rate and the temporal duration of water flow that may be proportional to the likelihood of bringing life to greater depths. A system with combined seismic pushes with thermal forces driving longer temporal, continuous water flow and higher permeability zones contribute to moving biomass to greater depths.

Seismic activity in mining operations is monitored and reports are available from Kopanang mine during the period between November 2012–January 2014 (Fig. S1). This partly anthropogenic seismic activity may raise criticism that deep mine research represents an artificial rather than a natural hydrogeological system, and is thus artificial. Deep subsurface research therefore studies an *augmented reality* not an *artificial reality*. Mining activity can change the natural state of a hydrogeological system in two ways. First, open boreholes (that are not valved or closed) and mine dewatering can potentially produce a more continuous and higher flow rate and volume of fracture water migrating from shallow/surface water sources compared to a natural system. However, in a comparable natural system (for example, if the fault/fracture running from the Vaal river to our subsurface sample point was not part of a mine) a seismic event that increased the length and aperture of a fracture/fault system would increase the hydraulic conductivity of shallow/surface water migration to the deeper subsurface as a discreet event on a more defined, non-continuous timescale. Second, additional seismic activity is created but, as shown from historical databases, South Africa had recorded seismic activity occurring long before mining activity began. The influence of mining activity on deep mine research must, therefore, be considered as an agent of augmentation of an existing state rather than an artificial one.

No Insecta-related RNA reads were identified but transcripts related to the flatworm *Stenostomum* was identified in the 2012 sample from Beatrix. *Stenostomum* has also been identified in the fracture fluids of Kopanang Mine^4^. No full length eukaryal 16S rRNA gene sequences were assembled using the EMIRGE pipeline but 6 unique 16S sequences related to 4 distinct classes of bacteria (Betaproteobacteria, Gammaproteobacteria, Deltaproteobacteria, and Anaerolineae). The longest contig related to degradation enzymes that we were able to assemble was a 254 nt long peptidase M42 family protein in both BE326 (2012) and TT109 (Supplementary Data 1). The 2011 sample from BE326 also contained a 141 nt read that had 100% alignment with the 254 nt transcript. Notably, cellulases belong to the M42 family of proteins and the closest sequenced genome hit to our assembled PEG was a “cellulase” from “*Ca*. Methanoperedens sp. BLZ1” (GenBank ID: KPQ42527.1; Amino Acids: 174–256). “*Ca*. Methanoperedens” is a methanogen that has been identified in both BE326 and TT109 at relatively low abundances (<0.5% of microbial community)^21^. Although we cannot say that there is a definitive lack of cellulase activity in FI88, the failure to detect 16S rRNA gene transcripts related to algae, plants, or diatoms combined with the lack of cellulase related transcripts in the FI88 metatranscriptome suggest that the presence of algae and/or diatoms is minimal. Furthermore, in instances where eukaryal and cellulose-degrading transcripts were identified, they were underrepresented in the metatranscriptomic library which in agreement with the low eukaryote encounter rate observed in our other experiments.

Chlorophyta (green alga), were recovered from two mines: Tau Tona: *Chlorella* and *Mesotaenium*, Star Diamond: *Chlorella, Mesotaenium* and *Crucigenia*. *Chlorella* is a cosmopolitan, single-cell green algae found in fresh water, salt water, and soil. It is spherical in shape, about 2 to 10 μm in diameter, and is without flagella^22^. *Chlorella* species can form survival spores^23^ and heterotrophy has been demonstrated^24,25^. Several *Chlorella* species have been found in extreme habitats^26^ and in one experiment survived 548 days in low Earth orbit exposed to conditions in outer space^27^. *Chlorella* has a well-established record for symbiosis with different eukaryotes including protozoa, and the invertebrate metazoan; Porifera, Plathyhelminthes and Coelenterata^28^. *Chlorella* species have been reported in the Republic of South Africa (RSA)^29^.

*Mesotaenium* is an unicellular green algae (Chlorophyta) with short, straight, cylindrical cells. The cells are solitary or aggregated within common mucilage to form irregular colonies. *Mesotaenium* is widely distributed but some species specialise in extreme habitats. *M. berggrenii* is restricted to very cold water and ice^30^. *M. testaceovagina* has only been found in seepages^31^. Heterotrophy (Mixotrophy) has been demonstrated in *Mesotaenium*^32^. Survival in extreme conditions via a vegetative stage is known^33^. No reports were found on brackish or saline habitats only reports of freshwater and peat bogs.

*Crucigenia* is a widespread freshwater algae (Chlorophyta) but has been reported from saline waters as well^34^. Spores have been reported^35^. *Crucigenia* species have been recovered from extreme habitats^36^. Resting spores have been described for *C. rectangularis*^37^. *Crucigenia* have been reported in RSA before^38^.

The discovery of viable green algae in water with residence times of thousands of years, collected with the same aseptic protocols as for bacterial/Eukarya before^1–3^, is surprising. However, another link, albeit a tenuous one, between mines and the alga *Mesotaenium*, was published in 1973^39^. Samples derived from a heavily polluted mine dumping site in the Gauteng Province, Klip river (Tributary of Vaal river) contained *Mesotaenium*^39^. Remarkable further is that in a list of all desmids reported in Southern Africa between 1855–2009 no *Mesotaenium* species are reported reliably anywhere else in RSA^40^.

Although the Tau Tona borehole was not valved, air contamination is unlikely as two samples taken on different dates only one had algae what would not be expected if the borehole had been colonized by algae. Furthermore it is difficult to imagine a 2–10 micrometer cell being able to move against a water flow of 0.33 L/min. The main question is how to explain the survival of green algae, for potentially millennia, in an absolute dark, hot environment. Two alternative metabolic pathways could be possible with green alga: heterotrophy or spore (resting stage) formation. Heterotrophy has been demonstrated for the three species recovered in the two mines. However the literature does not contain reports of green algae surviving in permanent dark conditions for thousands of years using heterotrophy. Most of the reports with dark adaptation concern the arctic winter where several species of *Chlorella* and *Mesotaenium* have been recovered^30,36,41^. Unless one would assume adaptation to long duration heterotrophy capability (for which we have neither supporting arguments nor evidence) we consider heterotrophy as a survival strategy highly unlikely as an explanation since the number of cells/ml found are low arguing against a blooming colony. The subsurface extreme conditions would more likely elicit a spore forming response from the alga recovered, a capability all three genera identified possess. The resistant cell walls of some of these taxa can survive degradation for thousands to millions of years, and these have been used by geologists for palaeo-environmental interpretations^42^. However the literature does not contain much information as to how long algal spores remain viable. The longest viability reported we found was for desmids and the calculated the maximum survival capability of these spores to be 262 ± 28 years in lake sediments^43^. Furthermore it has been reported that temperature also plays an important role in dark survival, with increasing temperatures causing a reduction in long-term cell viability but anaerobic conditions are favourable for long duration viability^42^. Algae colonize caves in many limestone or dolomite regions where fractures or underground streams form underground pockets or large caverns, including *Chlorella miniata* (for review see Wehr, 2003^44^) but in these cases there is always a minimal source of light into the cave.

Barring adaptation, both survival strategies do not adequately explain the observation of the algae recovered by neither mere heterotrophy nor spore formation itself. The only explanation that could marry the algal recovery in older fissure water with the age of the fissure water in Tau Tona is that the fissure water in Tau Tona is not homogenous and represents a mixture of paleometeoric water (2919–5165 yr) and recent meteoric recharge. In Tau Tona fissure water the ^3^H concentrations were within 2 s.d. of the detection limit^1^. If a more recent influx of meteoric water >80 yr occurred carrying algae it would have a negligible concentration of ^3^H (depending on mixing ratio) and still leave potential viable algae behind^4^. Additionally, the fissure water from Tau Tona is saline (14 g/L)^3^ and we could not find references in the literature of *Mesotaenium* species adapted to brackish or marine conditions. Furthermore the number of algal cells/mL recovered were low (also in the saline Tau Tona water) supporting a subdued population rather than a thriving one^45^.

We propose that the algae recovered are accidental, unadapted residents rather than a thriving, adapted indigenous population. The discovery of these alga fits the here proposed mechanism of a non-selective force that would transport organisms small enough to pass through a fault.

In Evander gold mine, Driefontein gold mine and Star Diamond mine three instances of Insecta were found in fracture water in three different mines in a period spanning 5 years by two different persons using three different methods.

In 2007 when sampling a slow flowing fissure (no borehole) at −1.7 km at Evander mine using an open plastic bottle a water beetle flowed with the fissure water into the bottle. The beetle (SVideo 7) was subsequently identified based on its size (2 mm) and the dark brown elytra with yellow markings; distinct furrows running parallel and close to the midline almost to the tip of the elytra (Friday, 1988) as *Hydroglyphus pusillus* (Fabricius, 1781) (Arthropoda; Dytiscidae; *Hydroglyphus* Motschulsky, 1853) a small cosmopolitan predacious diving beetle. The species is reported as a good flyer, pioneering the colonisation of new open water sources^46^. On the surface its feeding ecology is carnivorous where several reports mention a voracious capability to decimate mosquito larvae sometimes acting in group to attack and devour one prey^47^. Field trapping experiments show that during flight the beetles recognize the open body of water by its reflection of polarized UV light as evidenced by using glass panes as small as 100 × 60 cm that mimics this reflection and is the main optical cue for water-living insects^48^.

Although the adults mainly move in the water column, the larvae are known to reside mainly on the bottom of open body waters^46^. After recording the discovery on video footage the beetle was initially considered a contamination find in 2007 therefore no DNA analysis was done and the sample was discarded after the beetle died 25 days after it was collected.

Beetles are the most numerous of all insects with more than 300,000 species described. They inhabit a wide variety of environments including caves, lava tubes, cracks and fissures in a massif, mesovoid shallow substratum in limestone or in shist, gneiss, granodiorites, basalts, quartzits, grits etc. Of the 40 families of the Order Coleoptera, 15 have species living underground totalling around 2000 species^49^. A case in point: in the Late Miocene – Pliocene, (10–5 million years ago, Mya), the interior of Australia underwent aridification. During this process hundreds of subterranean aquifers in calcrete limestone deposited along palaeo-drainage systems became biologically isolated and revealed that surface species of diving beetles (Coleoptera, Dytiscidae) took refuge in these subterranean aquifers during one or more periods of extreme aridity. This resulted in the evolution of one to three species of blind, wingless, de-pigmented endemic species per aquifer^50^. Between 2000 and 2012 a total of 99 new stygobitic beetle species have been described from 52 isolated aquifers^51^. More closely related Closer related to deep subsurface conditions is the discovery in Monte Conca Cave, Italy where microbial mats are the source of an autotrophic system in close correlation with the biological cycle of many species of living organisms found near the sulfidic spring. Some of them show typical troglobitic characteristics, three species of Dytiscidae beetles are able to survive in that sulfidic water in the cave^52^.

In particular, troglobionts often show a combination of regressive characters (e.g., loss of eyes and pigmentation) and constructive characters (e.g., enhanced sensory structures not based on light sensing, longer lifespan, larger eggs, lower metabolism rate) that evolved independently in different lineages in a cave environment. Loss of wings and fused elytra in some species^49,53^. The *Hydroglyphus pusillus* recovered from Evander mine does not exhibit any of the above regressive or constructive characters that we could test in the absence of a surface counterpart.

On 20^th^ February 2009 a Cornelius canister was filled with fissure water under stringent aseptic conditions at the same borehole (−1.0 km) that yielded Eukarya^1,4^ at Driefontein mine. Subsequent 18S RNA gene analysis revealed the presence of a sequence with 99% identity to *Anisochrysa* possibly *A. carnea* STEPHENS, 1836 (*Chrysoperla carnea*) the common green lacewing (Eukaryota; Metazoa; Ecdysozoa; Arthropoda; Insecta; Neuroptera; Chrysopidae). Cosmopolitan but mainly Holarctic. Lacewings pass through 7 stages in their life cycle: egg, three larval instars, the prepupal instar, pupal and adult. A female can lay between 400–500 eggs. Males die after 1–2 weeks, females live longer in warmer climates 3–4 generations per year are possible. Diapause is present. The development from egg to adult takes on average 69 days at 15 °C, 31 days at 21 °C and 25 days at 28 °C. Below 10 °C development arrests^54^. Adult are good flyers up to 40 km per night^55^. Adults (12–20 mm) feed on pollen, nectar and honeydew. Larvae (about 8 mm long) are ferocious predators injecting digestive enzymes in their prey; they feed on aphids, whitefly, spider mites, thrips, butterfly eggs and small larvae of other insects^56^. We found no records of reports in caves or that part of the life cycle would be fresh water linked.

In 2012 at Star Diamond at a depth of −640 m the dorsal thorax shield (scutellum) was recovered from borehole fissure water to which a Eukarya filter (SFig. 2) was aseptically attached. No other insect parts were found nor was it possible on the basis of the scutellum to identify the species taxonomically. Since the filter was attached for 6 weeks it is unknown whether the insect was trapped alive, died and its remains digested by bacteria or whether the scutellum was a single piece being transported in the fissure water. No DNA analysis was attempted on this single chitin piece.

The discovery of three Insecta from depths ranging from −640 m to −1.7 km is enigmatic at best. The case of *Hydroglyphus pusillus* is the most difficult as it was collected with flowing fissure water in an open bottle at a non-drilled fissure in the rock face making this find sensitive to criticism that it is an external contamination. Although as mentioned before Dytiscidae have a solid proven record of colonizing subsurface aquifers. This leaves broadly two possible explanations for the origin of *Hydroglyphus pusillus*. Either it is endemic to the fissure water and came all the way from the surface or the beetle entered the mine via the main shaft in one way or another and settled in the fissure at −1.7 km as an external contaminant. The possibility that the beetle was transported into the mine via the service water used for cooling equipment and drinking is highly unlikely considering the severe bleach treatment this water receives before it is cooled and pumped into the mine and evidenced by our failure to even extract useable DNA from service water in mines on several occasions because of this chemical treatment^1–4^. Even when the air current in the main shaft would be advantageous for ‘sucking’ flying insects in, in general flying insects in mine corridors are extremely rare to absent. For example in Kopanang the vent is downcast down the main shaft to 47 level, from 47 to 44 level and then up to 42 level (sampling site is at 41 level) but no flying insect were recorded during our sampling. Additionally the specimen recovered showed none of the regressive characters associated with long term adaptation to the subsurface. No depigmentation, fusing of elytra was observed. Although this does not always occur in troglobytes it argues against a long term adaptation in the subsurface. However there are two observations that should be noted as well. Supplementary video SVideo 7 shows *Hydroglyphus pusillus* in a 50 mL Falcon tube to which a small amount of biofilm was added, hours after it was collected from the mine. At time intervals 1 min 05 s to 2 min 25 s and again at 2 min 56 s to 3 min 08 s the beetle actively visits the small amount of biofilm. Although the video does not allow determining whether the beetle actively fed on it or not, it is worth noting that the beetle survived an additional 25 days in this Falcon tube without any change of water or addition of food. Taking into account that this species is known as predator on mainly mosquito larvae and other small invertebrates and that subsurface fissure water is chemically different than pond water it does show adaptation has taken place in this specimen and that it can survive in fissure water at −1.7 km, otherwise its survival in the subsurface and the Falcon tube cannot be explained. Considering the above we cannot prove *Hydroglyphus pusillus* is endemic it could be an accidental arrival but the video does show that this specimen is comfortably able to survive the extreme conditions and with a different food source at −1.7 km.

The discovery of DNA from *Anisochrysa* sp. from a Cornelius canister filled with 12,000 yr old fissure water at −1.0 km at Driefontein is even more enigmatic. Here the sample was taken under very stringent aseptic conditions from a closed valve, only opened to take samples. External contamination is not a possibility in this case. Compounding the interpretation are the data that show the closest related species based on the DNA sequence is *A. carnea* a species that does not have a part of its life cycle in water. *A. carnea*, however, does have a demonstrated ability for diapause to survive harsher conditions^54^. Adults feed on nectar, pollen and honeydew, larvae are carnivorous on aphids, whitefly, spider mites, thrips butterfly eggs and small larvae of other insects. Barring adaptation for other feeding habits for which we have neither arguments nor evidence, it is not a diet availability to be expected in the subsurface. We believe the most likely explanation to be diapause stages that were transported to the subsurface from a surface source. Although the ^14^C age of the fissure water at Driefontein is 10,000–12,000 years old at the time of the sampling, this age makes it difficult to comprehend a diapause stage remaining intact for that length of time. Tritium analysis of the same sample however, showed that not more than 3% of the fissure water can be post 1980^1^. This more modern influx could be the source of the insect DNA and could explain why DNA detection was still possible. We believe *Anisochrysa* sp. is thus an accidental arrival.

In Star Diamond the discovery of a scutellum (SFig. 2) at −640 m was done using stringent aseptic conditions during sampling excluding external contamination. In the absence of other insect parts this can only be interpreted as the result of transport of this scutellum in the fissure water from a higher up source.

In conclusion two of the three Insecta (*Anisochrysa* and the scutellum) are accidental arrivals through the fissure water. There is no supporting evidence that *Hydroglyphus pusillus* is indigenous. It is most likely an accidental arrival as well (source unknown), but the recorded video shows that adaptation by this species is real and possible and that it was able to survive the different fissure water chemical and dietary conditions for at least 25 days and probably longer.

Based on geological, water chemistry and biological composition data we have demonstrated that surface freshwater Eukarya enter the deeper subsurface via a water channel and not terrestrial soil. This confirms and explains previous results^4^ that showed a near total absence of terrestrial soil Eukarya in subsurface fissure water samplings over a period of 10 years. The mechanism proposed here is non selective and might occasionally transport Eukarya to the deeper subsurface fast. This latter possibility also explains why the predominant Eukarya species recovered are already known species from the surface and the majority are well known opportunistic species able to survive a wide range of habitats^4^. The non-selective nature of the proposed seismic mechanism does explain the discovery of algae and Insecta, nearly all collected under stringent sterile conditions, in the deep subsurface. A major question remains whether the discovered Eukarya found up to −3.8 km^1^ deep have over time evolved into new species able to cope with even harsher conditions deeper in the subsurface. The discovery of a new nematode species *Halicephalobus mephisto*^1^ in 2011 at a depth of −1.3 km with no known surface population to date, shows that this is a possibility. Finally, as seismicity is a universal phenomenon and occurs on several planetary bodies in our solar system^57–59^. In particular there is ample evidence for past seismic activity on Mars when conditions for life were more favorable than today^57^. Under present day conditions on Mars predictions are that magnitude 2 seismic events still occur every 34 days and magnitude 7 events every 4500 years^59^. If life existed on Mars in the past, seismic activity may have transported life in to the deep subsurface long before the planet became inhospitable on the surface.

## Methods

### Sampling of borehole fractures

The Eukarya reported here from Kopanang mine were collected and reported separately before^4^. The river sampling and comparison was also completed at that time. Data from Zondereinde mine was from a sampling carried out and reported earlier^1^. The aseptic techniques and extensive controls were detailed in those reports^1,4^.

### Vaal and Bierspruit river sampling

The Bierspruit river sampling was performed on 06/09/2012 at 24°40′54.20″S 27°19′18.29″E, elevation 912 m. This was approximately 13 km upstream of the place where the fault that feeds the borehole intersects the Bierspruit river. The Bierspruit water level was very low at the time of sampling and the little water available was in a cul de sac. The sample was taken where the water edge met the border which was still moist as the water level had been declining (SFig. 3). A dead cow partially submerged in the water of the Bierspruit caused a brownish organic pollution. The sample was collected in a bucket and was approximately 5 L in volume water and mud mixed.

The Vaal river sampling was done on two separate occasions on 11/12/2013 at (Fig. 1) 27°00′36.41″S 26°41′52.45 E just of the Road 30 besides the Orkney bridge on the North West Province side of the river. Five 0.5 L bottles were taken 4 underwater mud adjacent to the river bank and one moist bank soil. Because the first sample was negative for similar Eukarya as found in the borehole, a second sample was taken on 28/05/2013 at 27°00′34.86″S 26°41′53.72 E on the other side of the Orkney bridge in the North West Province side of the Vaal River of where the previous sample was taken. Because the water level had subsided in intervening months, 8 sterile bottles of 0.5 L each were taken in a transect from moist bank to under water (SFig. 4). Both samples were collected approximately 5 km downstream from where the fault that feeds the Kopanang borehole intersects the Vaal River. On both samplings approximately 10 liters of river water was collected as well. For bacterial analysis planktonic samples were taken in sterile glass bottles. Samples were transported to the laboratory within 3 hours and allowed to settle in transparent 5 L flasks that were continuously aerated at room temperature thus ensuring a normal day/night regime. Using a stereomicroscope individual Eukarya were picked out using a flame sterilized platinum needle and processed for DNA extraction, light microscopy and/or SEM as appropriate.

### Eukarya isolation, maintenance and crosses between nematode strains

#### Isolation of Eukarya

Approximately 100 g of river mud was deposited directly in a Petri dish and flooded with river water in triplicate (nine plates total per sampling site) and the Eukarya were allowed to crawl out/revive. This allowed the Eukarya to multiply in sufficient numbers to observe them. Petri dish content was checked at least twice daily in the laboratory using a dissecting microscope in the days immediately after arrival in the laboratory. This was done to ascertain the well-being of the Eukarya. If it became obvious that Eukarya were not thriving (that is, increased transparency of the gut and loss of motility when appropriate) they were isolated and processed for SEM/DNA extraction, this to avoid losing this data. All attempts were made to keep Eukarya in culture.

#### Crossing experiments

The best way to determine if two morphometric and genetically identical species are indeed the same species is mating. To test whether the *Poikilolaimus oxycercus* populations from the Vaal river and the Kopanang fracture water could interbreed and produce viable offspring, males and females of both populations were mated. A previously described method^60^ was followed to facilitate comparison with one exception; the mating was done in fracture water with biofilm as food and not on agar plates. To mate virgin worms, two 3-day-old *P. oxycercus* larvae were picked and isolated to guarantee virginity before being used for mating experiments. When sexual morphologies could be determined, female–male pairs were left undisturbed and brood size, egg-hatching success and the life span of individual females were recorded. Males were left on the plate for as long as the oldest progeny was still clearly smaller than both parents. Three males were incubated per female at 20 °C for 5 days^60^. Mating plug structures on females were used as indication of successful mating. Intra-strain matings were done between the Vaal river *P. oxycercus* and Kopanang *P. oxycercus*. Additionally matings between *P. oxycercus* from Driefontein gold mine^4^ and Kopanang and Vaal river *P. oxycercus* were also done.

## DGGE

### analysis of PCR Products of ribosomal DNA fragments from Vaal river bacteria

The amplified16S rRNA gene fragments(600–800 ng) were separated on 7% (w/v) polyacrylamide gel with aurea/formamide denaturing gradient ranging from 40 to 60%. Electrophoresis was performed in 1X TAE buffer(40 mM Tris, 20 mM acetic acid, 1 mM EDTA; pH 8.3) at a constant voltage of 100 V at 60◦C and run for16h. Gels were stained for 40 min in 1X TAE buffer containing Sybr SYBR® Gold Nucleic acid stain (Molecular Probes, Thermofisher Scientific) and visualized with UV radiation by using a Gel doc XR and the Quantity one 4.6.7imaging software (Bio-Rad).

Sequencing reactions were performed with the ABI Prism^©^ Big Dye terminator^©^ V3.1 cycle sequencing ready reaction kit, and data were collected on an ABI 3130XL genetic analyzer (Applied biosystems). The sequences obtained were analysed by using Geneious 4.8.5 and compared with public DNA database sequences using BLAST on the GenBank nucleotide database (National Center for Biotechnology Information). The sequences were checked for chimeras using Bellerophon^61^. Sequences were managed and aligned by RDP. For comparison between bacteria from the Vaal river and the Kopanang fracture water the bacterial content of the fracture water at Kopanang mine was used as reported before^4^.

### Extraction and amplification of DNA from Eukarya

PCR amplification of 18S rRNA genePCR amplification of 18S rDNA fragments was performed using primer sets: EukA (5′-AACCTGGTTGATCCTGCCAGT-3′) and EukB (5′-TGATCCTTCTGCAGGTTCACCTAC-3′) and Nem18S(F) (5′-CGCGAATRGCTCATTACAACAGC-3′) and Nem18S(R) (5′-GGGCGGTATCTGATCGCC-3′)^62^. A standard reaction volume was 20 ml contained 1x concentration of standard Taq buffer (New England BioLab) (including 1.5 mM MgCl_2_), 0.5 mM of each primer, dNTPs at a concentration of 0.2 mM for each nucleotide, 0.025 units per ml Taq DNA polymerase and 10 ng of extracted DNA template. The thermocycling conditions used for eukaryal and nematode PCR reactions were as follows: an initial denaturation at 94 °C for 5 min; 35 cycles of amplification (94 °C for 30 s; 54 °C for 30 s; 72 °C for 1 min) and a final extension at 72 °C for 10 min^24^. The thermocycling machine used was a PXE 0.2 Thermal Cycler (Thermo Electron Corporation).

The amplified DNA fragments were sequenced using the ABI Prisma¨BigDyea¨ terminator V3.1 cycle sequencing kit (Thermofisher scientific), and data were collected on an ABI 3130XL genetic analyzer (Applied biosystems). The quality of ABI files retrieved using FinchTV software was evaluated and the sequence reads were assembled using CodonCode Aligner software (CodonCode Corporation, Dedham, MA, USA). Overlapping reads or contigs that represent the consensor regions of DNA were aligned using ClustalW (http://www.ebi.ac.uk/Tools/msa/clustalw2/). BLASTN analysis of the DNA database was used. Sequences were compared with the Nucleotide collection (nr/nt) database and ptimized for highly similar sequences (Megablast).

### Metatranscriptomic sequencing of fracture water

The presence of eukaryotes in the South African subsurface has been well established^1–4^ but molecular evidence of these microorganisms is limited. Here, we applied metatranscriptomic sequencing of fracture water from 3 different mines (TT109, FI88, BE326 from 2011 and 2012) to assess the activity of eukaryotes in subsurface samples. RNA was collected and sequenced according to Magnabosco *et al*.^63^.

### Sequence analysis

BLASTn databases were generated from the Silva Euk SSU nr database (https://www.arb-silva.de/projects/ssu-ref-nr/) and complete Silva SSU database (containing sequences from all 3 domains of life; https://www.arb-silva.de/projects/ssu-ref-nr/). Transcripts related to rRNA were downloaded from MG-RAST for all samples and blasted against the Eukaryote-only database (max e-value of 1e-10). Sequences that contained a positive hit to the Eukaryote-only database were then blasted against the complete SSU database. Sequences with an e-value less than 1e-50 and best hits relating to Eukaryotes were used in downstream analyses. This threshold was selected as sequences with an e-value less 1e-50 exhibited a relatively consistent consensus taxomony. Based on up to 5 annotations with the lowest e-values, the last common ancestor for each sequence was determined. The rRNA assembler EMIRGE^64^ was also used to assemble eukaryal rRNA sequences from the metatranscriptomic datasets. EMIRGE uses an iterative mapping method to perform rRNA assemblies and, thus, 3 reference datasets were used (Silva SSU, Eukaryote 18S from^4^. Finally, protein encoding genes (PEGs) of chitinase, pectinase, and cellulase were assembled from the metatranscriptomes using a targeted assembly pipeline^63^.

### Seismic *in situ* experiment

The *in situ* seismic experiment intended to show the short and long term effect of closing and sudden reopening of fracture water flow in boreholes on the biofilm. Two types of experiments were carried out. 1) A borehole that had been visually (camera footage) checked for copious biofilm growth was closed for 6 weeks after which it was reopened and visually checked again to evaluate the effect of fracture water flow stop on biofilm growth. This simulated the closing of a flowing fracture after a seismic event. The borehole was then allowed to flow again freely and rechecked 6 weeks later to visualize the re-colonization of the biofilm. 2) Flowing boreholes were closed off using a rubber plug, after a camera was installed inside. The fracture water was allowed to settle and the borehole was reopened in one move mimicking a seismic event creating a new fracture. The camera recorded the biofilm behaviour. Samples for water chemistry were taken periodically as was collecting of outflowing biofilm analysed. These experiments were carried out at different mines where big enough unobstructed boreholes were available. Experiment 1was carried out at Star Diamond mine, experiment 2 was carried out at Star Diamond, Kopanang and Finsch diamond mine.

## Supplementary information

Supplementary information

supplementary data videos

LaTeX Supplementary File

## Data Availability

Sequence information has been deposited at GenBank under accession numbers MF437018, MF437019, MF542306-MF542310, MF511810-MF511818, PRJNA308990 and PRJNA517956.
