# Supplementary material for: New ecosystems in the deep subsurface follow the flow of water driven by geological activity
Source: Sci Rep. 2019 Mar 1;9:3310. doi: 10.1038/s41598-019-39699-w (PMC6397172; doi:10.1038/s41598-019-39699-w)
Supplement: Supplementary file 1 — Supplementary information [file 41598_2019_39699_MOESM1_ESM.docx]

**SUPPLEMENTARY INFORMATION**

New ecosystems in the deep subsurface follow the flow of water driven by geological activity.

G. Borgonie^*1^, C. Magnabosco^2+^, A. García-Moyano^3+^, B. Linage-Alvarez^4+^, A.O. Ojo^4^ , L.B. Freese^5^, C. Van Jaarsveld^6^, C. Van Rooyen^4^, O. Kuloyo^7^, E.D. Cason^4^, J. Vermeulen^4^, C. Pienaar^5^, E. Van Heerden^8^ B. Sherwood Lollar^9^, T.C. Onstott^10^ & S.O.C Mundle*^11^

1 Extreme Life Isyensya (ELI), PB 65, 9050 Gentbrugge, Belgium.

2 Flatiron Institute Center for Computational Biology, Simons Foundation, 162 5^th^ Ave., New York, 10012 New York, USA

3 Centre for Applied Biotechnology, Uni Research AS Thormøhlensgate 55

N-5008 Bergen, Norway.

4 Department of Microbial, Biochemical and Food Biotechnology, Swot Street 9300

Bloemfontein, Republic of South Africa.

5 AngloGold Ashanti Kopanang Mine, Private Bag X5010, Vaal Reef, North West 2621, Republic of South Africa.

6 Northam Platinum Ltd., Zondereinde Division, Farm Zondereinde 384KQ, District Thabazimbi, Limpopo Province, Republic of South Africa.

7 Department of Geoscience, University of Calgary, 2500 University Drive

Northwest Calgary, Alberta, Canada T2N 1N4.

8.Biosaense,Walter Sisulu 5, Bloemfontein 9031, South Africa

9 Department of Earth Sciences, University of Toronto, 22 Russell Street, Toronto, Ontario, Canada M5S 3B1.

10 Department of Geosciences, Princeton University, B79 Guyot Hall, Princeton, 08544 New

Jersey, USA.

11 Great Lakes Institute for Environmental Research, University of Windsor, 401 Sunset Ave., Windsor, ON, Canada N9B 3P4.

**SUPPLEMENTARY METHODS**

**Sampling and decontamination methods.**

Samples for Eukarya were taken using similar techniques as described^1^ with the following modification. The filters were filled with the same rock from the borehole site. Small pieces of rock were collected, washed in 100% ethanol, rinsed in distilled water and put in the filter. This provided nutrients to the bacteria passing through these rocks allowing these to grow and serve as food for the Eukarya. As such we were able to execute longer-term filtering of the fissure water. Samples were taken by mounting a Margot-type expansion plug into existing boreholes. The expansion packer was fitted with a Delran manifold with Tygon tubing through which water flowed to a 38-mm-pore-size membrane filter housed in a cylindrical tube. The plug, manifold and tubing were washed, autoclaved and sealed before transport to the mine and quickly assembled and inserted into the boreholes. The borehole water flowed through the plug for several minutes to remove any surface contamination introduced during the insertion of the plug before the manifold and tubing was attached. Water was then allowed to flow through the manifold and tubing for several minutes before the sterile filter apparatus was attached. After the filter was attached, the water flowed through it to a plastic tube equipped with a small flow indicator. To prevent mine air from reaching the filter and to stop reverse contamination by air-borne Eukarya. The bottom of the cylinder holding the filter was fitted with a jagged edge, causing the water, once it passed the rock and filter and reached the bottom of the tube, to swirl, creating a water lock. The small flow indicator slowed the water as well creating a second water lock while maintaining a flow, high enough to prevent Eukarya from entering the filter from the bottom. The set-up was equipped with valves at the inlet and outlet, allowing one to isolate the filter apparatus from air before dismounting it from the packer for transport to the laboratory. A typical sampling event involved insertion of the plug and sampling manifold, attaching a Cole Parmer, 0.2 mm effective pore size, double open-end, high-efficiency, pleated polytetrafluorethyleen (PTFE) filter cartridge (<http://www.coleparmer>. com—EW-06479-52), 8 cm in diameter and 25 cm long. This filter was mounted in a 304 SS housing (Cole-Parmer, EW-01508-40) and autoclaved before connecting. Over-filling of two 1-litre amber bottles for ^3^H and ^14^C analysis and filling of several smaller-volume serum vials and falcon tubes for geochemical analysis. After collecting these samples, the Eukarya-trapping rock filter apparatus was attached to the manifold and left while the borehole water flowed through it. After the incubation period, the filter apparatus was disconnected and sealed, the plug and manifold removed and the valve (if present) closed. The Cole Parmer filter cartridge was emptied in the mine and frozen until analysis. All samples were then transported to the University of the Free State laboratory. To test for possible sources of eukaryal contamination

within the mine, readily available soil/water from puddles was collected with the anticipation that the soils would have far more Eukarya per gram than would the mining water, because the latter was chilled and treated with disinfectants. (1) Wet soil within the borehole cubby either from underneath or in the immediate neighbourhood of the borehole valve was also collected, the reasoning being that if Eukarya were present in this soil sample they may have originated from the borehole water when it flowed out or from the mining water when the borehole was first drilled and started living in the soil, despite the cooler and drier conditions. (2) Soil samples in the tunnel immediately adjacent to the borehole cubby were collected as a way of determining the prevalence of Eukarya within the mine tunnels open to ventilation and human traffic. (3) Wet soil under or near the mining water outlets was also collected. (4) For the Driefontein gold mine, a second Eukarya filter, filled with cotton as a retention substrate for bacteria as food for eventual Eukarya, was attached to a plastic hose and a total of 3,865,654 l of mining water was filtered. This was done since the use of inline filters is only suitable for shorter periods and is not reliable beyond 2 years of continuous sampling. In the case of the Kopanang gold mine, access was granted to the mining water system for filtration using the same filter set-up used for filtering fissure water (Cole Parmer filter). A connection was made at a water line located near the top of the Kopanang gold mine right after the mining water treatment plant. The mining water sample was filtered for a total volume of 50,400 l. Although it would have been ideal to filter the same amount of mining water as control as the amount filtered for Eukarya, this was practically impossible considering the large volumes of commercially obtained mining water. Here the filtering had to cease as a result of periodic maintenance work at the cooling station, making longer stretches of filtering impossible. At Driefontein it was not possible to let the mining water flow at the same rate as the borehole for such an extended period of time. Still the more than 3,865,654 l of mining water tested at Driefontein gold mine is the largest volume of mining water ever tested over an extended period, and we are confident that such a volume would have shown eukaryal contamination if it were present. The mining water at Kopanang mine was treated as identically as published before^1^, which resulted in the inability, then as now, to even extract usable DNA^1,4^.

.

**Site descriptions**

**The Kopanang gold mine (Village Main Reef previously owned by Anglogold Ashanti) 26°58’59.40" S 26°44’38.85" E.** The Kopanang mine is located in the Free State Province, 8 km East of Orkney. Kopanang exploits gold- and uranium-bearing conglomerates of the Central Rand Group of the Witwatersrand, the most important being the Vaal Reef (https://mining-atlas.com/operation/Kopanang-Gold-Mine.php). The sampled borehole was located at level 42, at 1.4 km below the surface in shaft #9. The borehole K7018 was drilled in 2007 from 42 BW2 9N xc at an inclination of -5 degrees to a depth of 154 m. The depth below datum is 1744.66 m. The intention of the hole was to drill for cover of the advancing development and to intersect water and or gas ahead of the face. The borehole was not sealed and flowed continuously at 3.15 l.min^-1^, the temperature of the water was 31.3 °C. A Cole Parmer 0.2-mm filter was installed from 11 December 2012 until 08 May 2013. A volume of 654,821 l of fracture water flowed over the filter. An identical Cole Parmer 0.2 mm filter as used for fracture water sampling was attached, just outside the plant on the surface, to the water used inside the mine for cooling to determine any outside sources of contamination. A total of 50,400 l flowed over this filter, which had to be removed prematurely because of maintenance work on the cooling plant, preventing longer flow time. Fig. 1 shows the fault underlying the Vaal river all the way to the borehole. The main fault material are cataclasites and ultracataclasites. The dip of the fault is in general very steep, 75 to 90 degrees, dipping to the South with a vertical depth of approximately -1320 m below surface. The zone thickness is approximately 3 m. Besides sampling this borehole was also used for the *in situ* seismic experiment.

**Zondereinde Pt Mine (Northam Platinum Ltd.) 24°49’48.69 S 27°20’28.38’’ E.** Zondereinde Pt Mine is located in the Transvaal Province within the 2.0 Ga Bushveld Igneous Complex (BIC) and the results on one water sample from this mine have been published previously^27^. The sampled borehole was located on the level 7 at 7 level foot wall drive west 1.7 km below the surface in the shaft #1. The dip ranges between 60° to the northeast to sub-vertical. This site was chosen because the borehole was fed by a fracture system that runs all the way to the surface to the Bierspruit river (Fig. 2) and exemplified a possible route for Eukarya to migrate down to the deep subsurface. The flow rate for the Zondereinde Pt Mine borehole was 8,640 L day^-1^ and the borehole was sampled only once for a total of 8,640 L. It was located in the Upper Critical Zone of the western lobe of the Rustenburg Layered Suite of the Bushveld Igneous Complex (BIC). The borehole was not sealed and flowed continuously. Although the Zondereinde borehole is half the depth of the Tau Tona borehole, fluids flowing from the boreholes were recorded to be 48°C. This is because the geothermal gradient of the BIC is 21.9°C km^-1^, twice that of the Witwatersrand Basin^28-30^.

**Evander Gold Mine (Harmony Gold Mining Company Ltd.) 26°27’20.57’’ S 29°4’15.76’’ E.** Evander gold mine is situated 8 km northwest from Secunda in the Mpumalanga Province. The Evander Basin is a tectonically preserved sub-basin outside the main Witwatersrand Basin and forms an asymmetric syncline, plunging north-east. It is structurally complex with a series of east-north-east striking normal faults. At the southeast margin of the basin, vertically to locally overturned reef is present. The only economic reef horizon exploited in the Evander Basin is the Kimberley Reef. The Intermediate Reef is generally poorly mineralized, except where it erodes the sub-cropping Kimberley Reef to the south and west of the basin. A single sample yielding a Coleoptera was collected in 2007 at shaft #8, level 13 at a depth of ∼1.7 km.

**Tau Tona Gold Mine (AngloGold Ashanti Ltd.) 26°21’21.29 S 27°24’10.12’’ E.** Tau Tona Gold mine is located south of Carletonville in the Gauteng Province and about 70 km southwest of Johannesburg. The sampling site was a nearly horizontal borehole located on level 118 at a depth of 3.4 km in the Witwatersrand Supergroup and intersected the Pretorious fault zone. The borehole was not sealed and flowed intermittently. This borehole was the site of the deepest metazoan ever found in 2011 and again in 2015^1-4,31^. The fracture water was sampled again in 2012–2013 in an effort to recapture the Monhysterid nematode discovered before. Subsequent analysis by the Institute for Ground Water studies at the University of the Free State, discovered Algae.

**Driefontein Gold Mine (Sibanye gold) 26°25’011.80" S 27°30’09.98" E.** The Driefontein Gold mine is situated some 70 km west of Johannesburg near Carletonville in the Gauteng Province of South Africa. The mine is located on the North Western Rim of the Witwatersrand Basin. It comprises eight production shafts that mine different contributions from pillars and open ground. Three primary reefs are exploited: the Ventersdorp Contact Reef located at the top of the Central Rand Group; the Carbon Leader Reef near the base and the Middelvlei Reef, which stratigraphically occurs some 50–75 m above the Carbon Leader Reef. It is a large, well-established deep-to-ultra-deep level Gold mine extending to level 50, the lowest working level, at some 3,400m below surface. It has been the subject of numerous previous studies^13,32-33^.

The borehole sampled was located in the intermediate pumping chamber at 1.0 km depth of #5 shaft at Driefontein Gold Mine (formerly known as East Driefontein Gold mine). The intermediate pumping chamber is a pump station for lifting water from the 3-km-deep mining levels to the surface and is also the location where water from the regional Transvaal dolomite aquifer is used to replenish the mining water lost during recirculation^29^. The borehole that was sampled was drilled in 1998, intersects the dolomite aquifer and was sealed with a valve. On February 20^th^ 2009 a Cornelius canister was aseptically attached to this borehole yielding in subsequent DNA analysis the sequence of a lacewing insect.

**Star Diamonds Mine (Petra Diamonds) 28°19’06.72"S 26°47’39.80"E.** This mine is located in Free State Province near The towns of Welkom and Virginia, some 240 km southwest of Johannesburg in the southern Witwatersrand Basin rim. The borehole sampled was located at 4 shaft, 15 level East, nr. 4 cross cut, at 640 m below the surface. The water was associated with fractures in a Kimberlite dike. The borehole was not sealed with a valve. An Eukarya trapping filter was aseptically attached for six weeks in 2012 and yielded the scutellum of a beetle. This borehole was also used for in situ seismic experiments.

**Beatrix Gold Mine (Gold Fields Ltd.) 28°14’ 24’’ S 26° 47’ 49.30’’ E.** Beatrix Gold mine is located near the towns of Welkom and Virginia, some 240 km southwest of Johannesburg in the Free State Province of South Africa. Geologically the mine is located along the Southern Rim of the Witwatersrand Basin. It consists of four operating shafts with levels at depths between 600 and 2,155 meters below surface. The fracture water from this mine has been studied previously^34^. The two boreholes, BH1 and BH2 are located on level 26 of #3 shaft, 1.3 km below the surface in a tunnel excavated in 2007. They are located in the Witwatersrand Supergroup, which at this location is directly overlain by 400-800 m of Carboniferous Karoo sediments^32,35-36^. Both boreholes were sealed with valves.

**Finsch Diamond Mine Petra Diamonds, Pty Ltd). 28°23’09.46’’S 23°26’39.66’’ E.** Finsch diamond mine, located near Lime Acres, is on the Ghaap Plateau 165 km west of Kimberley in the Northern Cape province. It is South Africa’s second largest diamond operation by production. Finsch is a classic diamondiferous Kimberlite pipe, which has a surface expression of around 17.9 ha. The country rocks consist of banded ironstones overlying dolomites and limestones, the pipe itself consisting of weathered Kimberlite to a depth of around 100 m with unweathered material beneath. The borehole was situated at -880 m in a horizontal position for unknown length. It was not sealed with a valve. A large horizontal borehole was used for in situ seismic experiment using a HD camera.

**Arthropoda collection**

Evander. A coleopteran was collected on 25/04/2007 in a corridor approximately 50 meters from the main elevator in on open 500 ml bottle at Evander mine at shaft 8 level 11 in Ventersdorp lava formation from a fracture in the wall at -1.7 km. Fracture water temperature was 22.5°C, pH: 8.93 conductivity: 1.86 mS. Upon return to the beetle was transferred to a 50 ml Falcon tube with the original fracture water and some biofilm and filmed (SVideo 7).

Driefontein. During microbiological water collection in December 2008 by Dr. Antonio Garcia-Moyano using a Cornelius canister, subsequent DNA analysis revealed the 18S rDNA sequence of a lacewing. The sample was taken at -1.0 km. This borehole has yielded several different species of Nematoda before^1-4^.

Star Diamond. A sterile Eukarya trapping filter was attached to a recently drilled borehole at -640 m in 2012. After recovery on the 38 µm filter mesh a piece of scutellum of a coleopteran was recovered (SFig 2). No other pieces of the cuticle were identified on the filter mesh.

**Algae collection**

During routine water chemistry analysis at the Institute for Ground water Studies (IGS) at the University of the Free State two occasions of samples containing Chlorophyta (green algae) were identified. Samples were formaldehyde fixed and evaluated using an inverted microscope, the samples were routinely discarded after analysis and thus no longer available to us for further analysis. Several other samples from different mines (Kopanang gold mine, Jeol gold mine, Zondereinde platinum mine, Finsch diamond mine) were also checked but were negative for algae.

Tau Tona. Sample TT118FW160413 collected under sterile conditions at -3.6 km was determined to contain two species of Chlorophyta (green algae): *Chlorella* 733 cells/ml and *Mesotaenium* 293 cells/ml.

Star Diamond. As above, a single sample collected at -640 meters in 2012 was determined to contain three species of Chlorophyta: *Chlorella* 1.977 cells/ml, *Crucigenia* 220 cells/ml and *Mesotaenium* 440 cells/ml.

**Video equipment**

The borehole/fracture camera system was assembled in-house at the University of the Free State using only of the shelf equipment with minimal modifications. Several different video cameras were used to make the footage presented in this paper. The smallest was a commercially available USB-powered Voltcraft BS-15 endoscope (Voltcraft, Conrad online, Belgium), which was lengthened in-house at UFS to 20m using a USB cable equipped with a repeater (brand unknown). This camera gave a 640x480 VGA live view recorded on the freeware recording program Splitcam (Splitcamera.com). Another camera having higher resolution but no live view and recorded on SDHC cards (Samsung) was the Roadhawk 720p HD camera (DCS systems Ltd, Essex, UK; waterproof -10 m). Except for the endoscope, the Roadhawk camera had no own light source. To provide sufficient light in the borehole several small or bigger flashlights (LEDLenser, Zweibrueder Optoelectronics GmbH, Solingen, Germany) were used depending on the diameter of the borehole. As these were not always waterproof, insulating tape was used to seal potential points of water entry.

This worked most of the time to keep the flashlights waterproof and operating. Depending on the size of the borehole and the length and type of the experiment, cameras, flashlights were mounted on a U- or V-shaped profile aluminium rail, which was connected to a 30-m-long metal spiral spring (WEBCO, Modderfontein, RSA) normally used to pull electrical wires in homes.

**Geochemical methods**

Temperature, pH, oxidation-reduction potential (ORP),

dissolved O_2_, conductivity, resistivity and total dissolved solids (TDS) were measured

on site using a Hanna HI9828 multiprobe. Salinity was measured on site using an ATAGO Pocket refractometer. Total iron, hydrogen sulphide, nitrite and dissolved oxygen content were determined by colorimetric analysis using CHEMet self-filling ampoules (CHEMetrics Inc., USA). Cation analyses were performed on filtered water samples using a DV ICP-OES (Perkin Elmer Optima 3000). Anion analyses were performed using ion chromatography (Dionex DX-120) with an Ionpac AS14 (4 x 150 mm) analytical column and an Ionpac AG14 (4 x 50 mm) guard column. The total organic carbon (TOC) and dissolved organic carbon

analyses were performed on unfiltered and filtered water samples, respectively, using the persulfate ultraviolet oxidation method and a Formacs Low Temperature TOC analyser (Skalar van Holland). The NH_4_^+^concentrations were determined using the Nesslerization method. The cation, anion, TOC, dissolved organic carbon and NH_4_ analyses were conducted at the Institute for Ground Water Studies at University of the Free State. Analyses of the δ^13^C and ∆^14^C of the dissolved inorganic carbon (DIC) for the water samples were carried out by AMS at the National Isotope Centre, Institute of Geological and Nuclear Sciences Ltd, Lower

Hut, New Zealand. Water isotope analysis was carried out commercially at the University of Waterloo, Waterloo, Canada. The ^14^C and tritium data were modelled previously^4^.[

**Scanning electron microscopy (SEM)**

Biofilm was fixed at 4 °C in 2.5% glutaraldehyde, washed three times for 20 min each in PBS (137 mM NaCl, 2.7 mM KCl, 10mM Na_2_HPO_4_ and 2 mM KH_2_PO_4_) pH 7.2. Post fixation was carried out in 1% OsO_4_ for 1 h at room temperature. Dehydration was achieved through a graded acetone series (20–40–60–80–100%) in 1-h steps followed by critical point drying^37^, 1995). After critical point drying, specimens on stubs with carbon discs were sputter coated with gold using a Bio-Rad (Microscience Division) Coating System (London, UK) and observed with a Jeol JSM 8440 SEM microscope.

**References**

27. Gihring, T. M. et al. The distribution of microbial taxa in the subsurface water of the Kalahari Shield, South Africa. *Geomicrobiol. J.* **6**, 415–430. <https://doi.org/10.1080/01490450600875696> (2006).

28. Omar, G., Onstott, T. C. & Hoek, J. The Origin of Deep Subsurface Microbial Communities in the Witwatersrand Basin, South Africa as Deduced from Apatite Fission Track Analyses. *Geofluids* **3**, 69-80. <https://doi.org/10.1046/j.1468-8123.2003.00050.x> (2003).

29. Durrheim, R. J., Spottiswoode S. M., Roberts M. K. C. & Brink A.v.Z. Comparative seismology of the Witwatersrand Basin and Bushveld Complex and emerging technologies to manage the risk of rockbursting. The Journal of The South African Institute of Mining and Metallurgy **105**, 409-416 (2005).

30. Zindi, L. in *Third International Platinum Conference ‘Platinum in Transformation'* (The Southern African Institute of Mining and Metallurgy). 375-386. (2008).

31. Takai, K., Moser, D. P., DeFlaun, M. F., Onstott, T. C. & Fredrickson, J. K. Archaeal diversity in waters from deep South African Gold mines. *Appl. Environ. Microbio.* **67**, 5750-5760, doi:10.1128/AEM.67.21.5750-5760.2001 (2001).

32. Lippmann, J. et al. Dating ultra-deep mine waters with noble gases and 36Cl, Witwatersrand Basin, South Africa. *Geochim. Cosmochim. Acta* 67, 4597–4619. 10.1016/S0016-7073(03)00414-9 (2003).

33. Moser, D. P. et al*.* Temporal shifts in microbial community structure and geochemistry of an ultradeep South African gold mine borehole. *Geomicrobiol. J.* **20,** 1–32. <https://doi.org/10.1080/713851170> (2003).

34. Lin, L. H. et al. Planktonic microbial communities associated with fracture-derived groundwater in a deep gold mine of South Africa. *Geomicrobiol. J.* **23**, 475-497. [ttps://doi.org/10.1080/01490450600875829](https://doi.org/10.1080/01490450600875829) (2006).

35. Onstott, T. C., et al. The origin and age of biogeochemical trends in deep fracture water of the Witwatersrand Basin, South Africa. *Geomicrobiol. J.* **23**, 369–414. doi: 10.1080/01490450600875688

36. Moser, D. P., DeFlaun, M. F., Onstott, T. C. & Fredrickson, J. K. Archaeal diversity in waters from deep South African Gold mines. *Appl. Environ. Microbiol.* **67**, 5750-5760. DOI:[10.1128/AEM.67.21.5750-5760.2001](https://doi.org/10.1128/AEM.67.21.5750-5760.2001) (2001).

37. Borgonie, G., Van Driessche, R. & Coomans, A. Scanning electron microscopy of the outer and inner surface of the buccal cavity of some Mononchida. *Fundam. Appl. Nematol.* **18**, 1–10 (1995).

**SUPPLEMENTARY TABLES**

**STABLE 1.** Bacterial composition of the Vaal water sample. There is no overlap with the data for the Kopanang borehole as detailed in previous research^4^

DGGE closest hit Accession no. % identity Taxonomic affiliation

sample

V1 Uncultured Cyanobacteria JN377916 99 Cyanobacteria river

V2 Uncultured bacterium JQ978313 99 Unclassified bacteria lake

V3 Uncultured Nitrosomonadaceae JN377924 100 Betaproteobacteria river

V4 Uncultured Anabaena sp. JN377927 99 Cyanobacteria river

V5 Uncultured Bacterium JN377933 100 Unclassified bacteria river

V6 Sphingobacterium sp. KM658454 100 Bacteriodetes soil

V7 Uncultured Aeromonas sp. HE981721 100 Gamma-Proteobacteria river

V8 Uncultured Bacteroidetes LN869913 96 Bacteriodetes river

V9 Uncultured Bacillus sp JX504062 99 Firmicutes river

V10 Uncultured Pseudomonas X504058.1 99 Gamma-Proteobacteria river

**STABLE 1.** Bacterial composition of the Vaal water sample. There is no overlap with the data for the Kopanang borehole as detailed in previous research^4^

|  | Depth (km) | Fissure water residence time^1^ (kyr) | Salinity^2^  (ppm) | Fungi | Algae/Diatoms | Ciliate | Amoebozoa | Metazoa |
| --- | --- | --- | --- | --- | --- | --- | --- | --- |
| TT109 | 3.1 | 16-21 | 296 | X |  |  |  |  |
| FI88 | 1.0 | 410 | 1282 | X |  |  |  |  |
| BE_2011 | 1.3 | >40 | 4473 | X | X |  |  |  |
| BE_2012 | 1.3 | >40 | 3586 | X | X | X | X | X* |

**STABLE 2.** Eukaryotes identified by BLASTn in the metatranscriptomic datasets. Taxa representative of the various types of eukaryotes in the various sites are represented by an X. ^1^Data adapted from^56^. ^2^Data adapted from^14^. *rRNA hits for *Stenostomum* (Platyhelminthes) previously identified also in Kopanang mine^4^.

**SUPPLEMENTARY FIGURES**


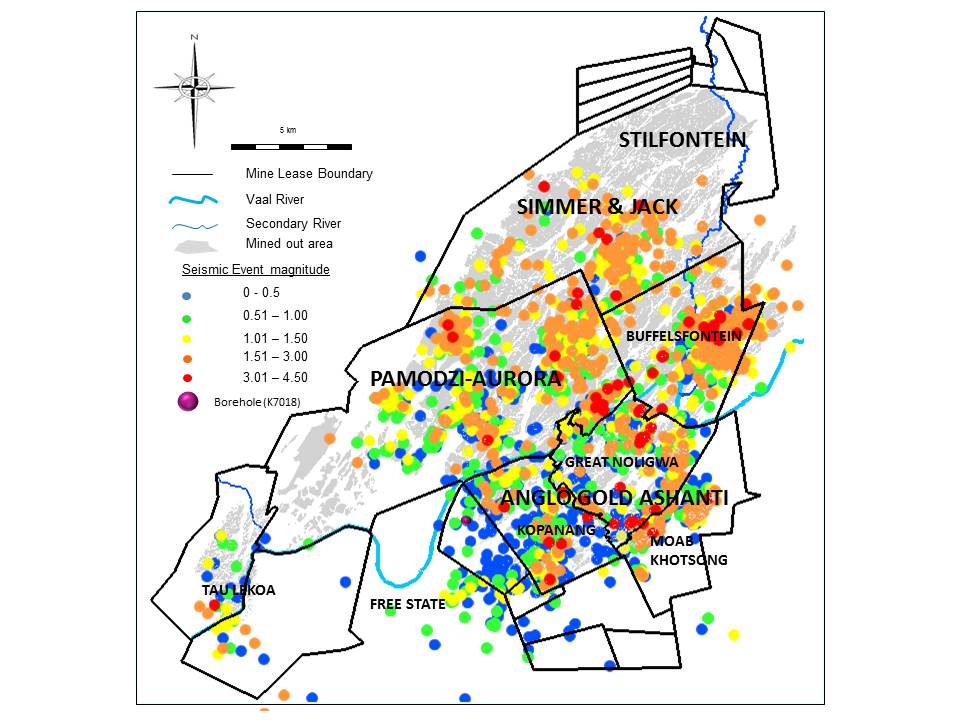


**SFigure 1. Map of seismic activity at Kopanang mine 2012-2014.** The map indicates that events are concentrated mostly around the areas where active mining is taking place, with minor events ahead of the mining fronts. Map courtesy of Kopanang mine. Scale bar 5 km.


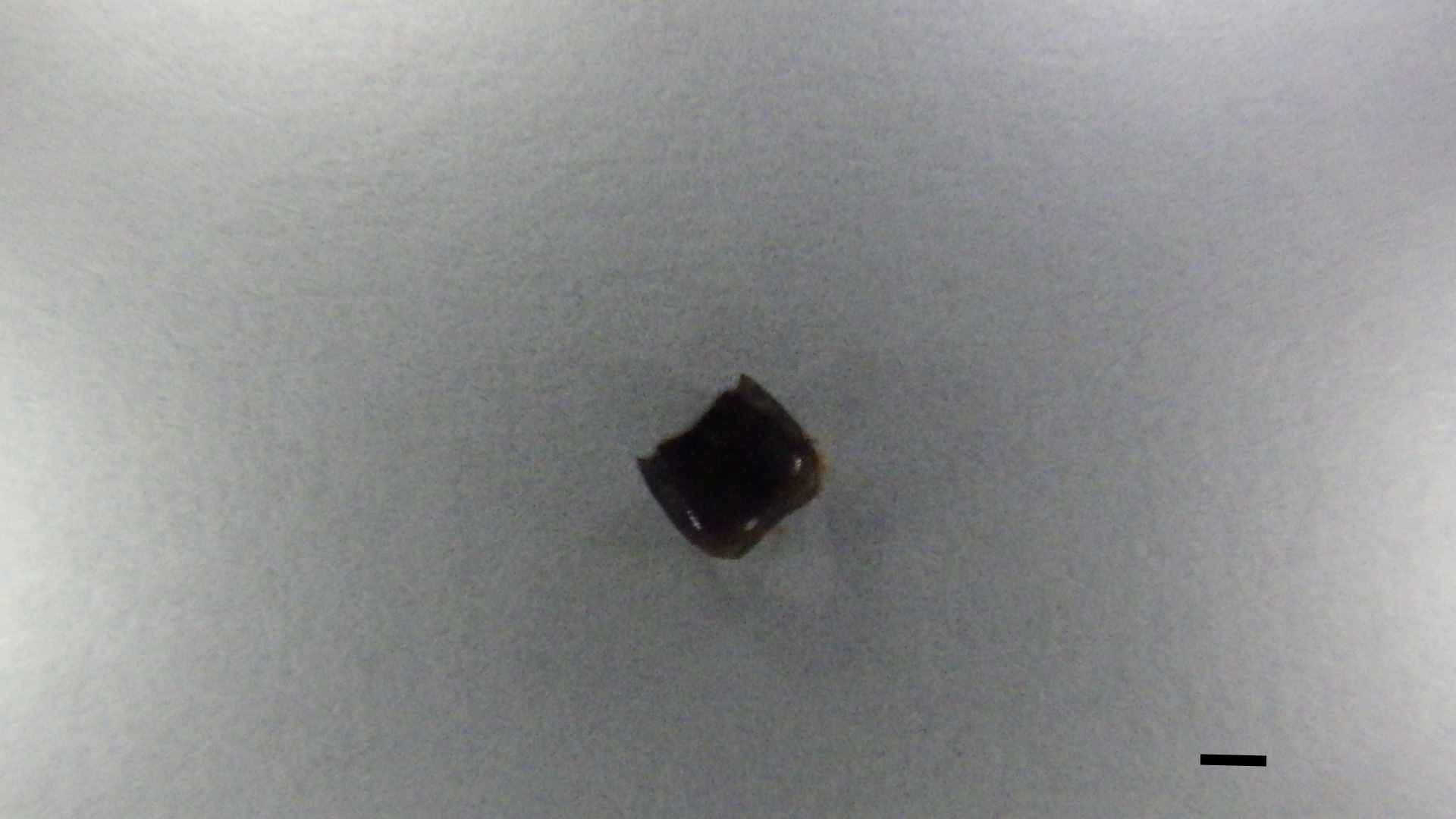


**SFigure 2. Scutellum of a unknown beetle.** Scutellum retrieved from an Eukarya trapping filter at Star Diamond mine at -640 m. Scale bar: 0.3 cm.


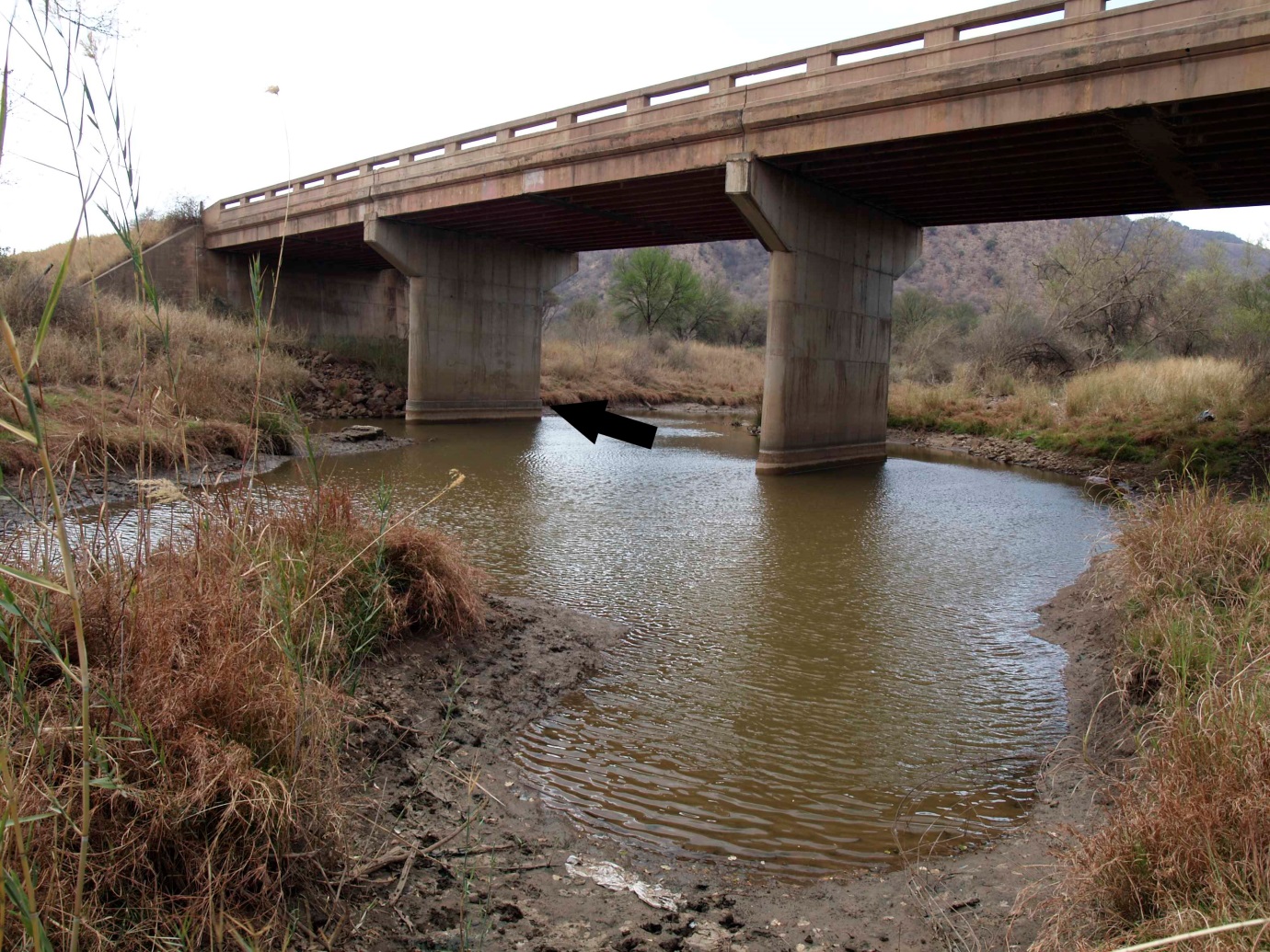
**SFigure 3. Sampling at the Bierspruit River.** 4.5 liter mixed sample of mud and water was taken (arrow) from the Bierspruit river at this location. The river level was very low and the carcass of a dead cow nearby was partially submerged and contaminating the water. Picture taken by G.B.


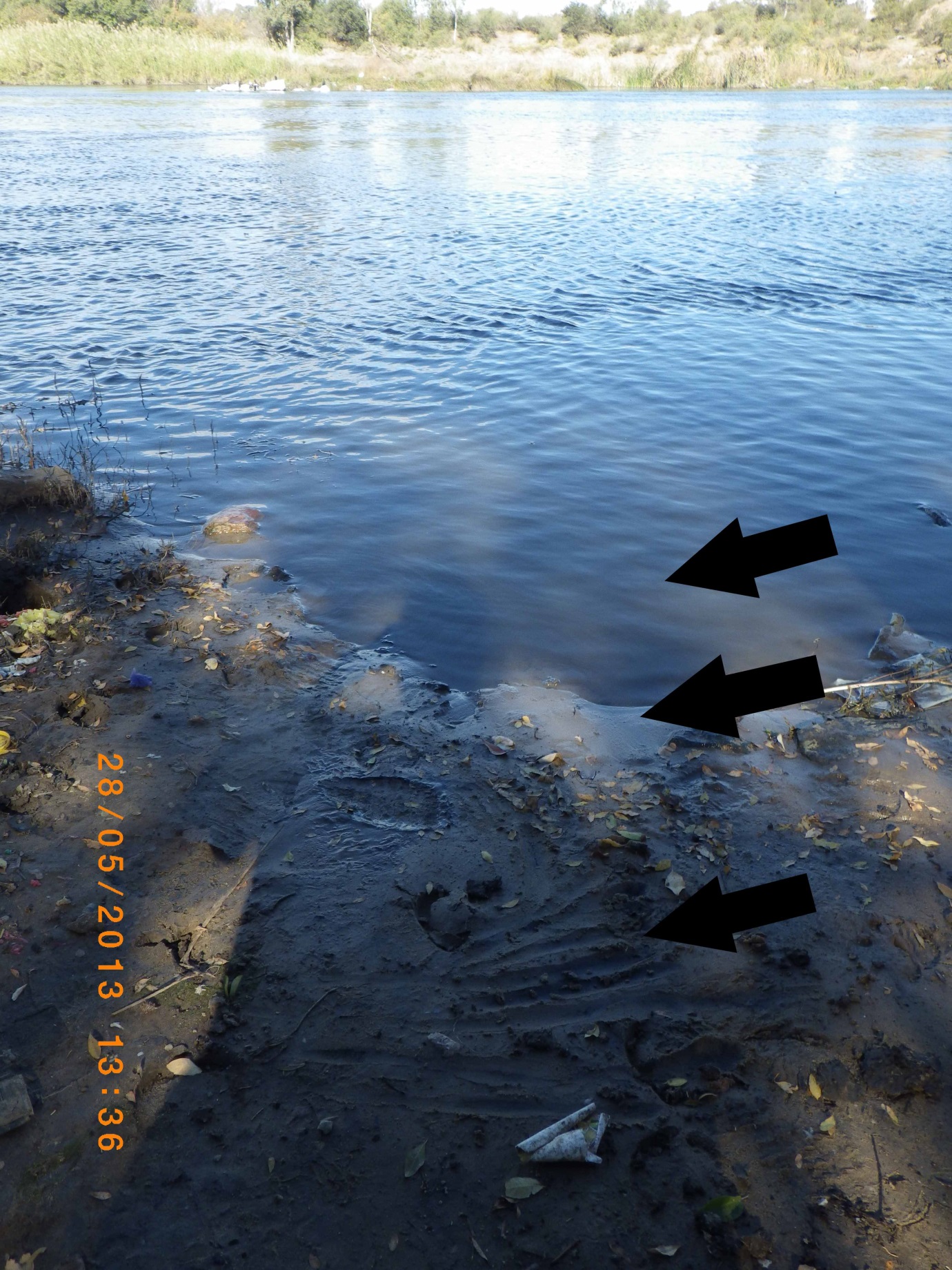


**SFigure 4. Sampling at the Vaal River near the Orkney bridge.** This is the site of the second sampling after the river level had subsided in comparison to the first sampling attempt. The sampling was executed in a transect from bank to into the river (arrows). A total of 15 L water and mud was collected at the Vaal River in two sampling events. Picture taken by G.B.

**VIDEO LEGENDS**

**SVideo 1. Seismic event simulation at Kopanang gold mine.** After that the borehole had been closed for 5 months, flow was reestablished and samples were collected in batches of 20 minutes for a total of 80 minutes. The second batch of 20-40 minutes yielded small pieces of biofilm with nematodes in suspended animation (dauer stage). The resolution was 1820x720 at 25 fps and recorded as an mp4 file. The file was converted to a MOV format (H264) and deinterlaced using WinX video converter 5.0.4 (Digiarty Software Inc, Chengdu, China) without any other changes.

**SVideo 2 Star Diamond Seismic event simulation.** After the borehole had been sealed for 6 weeks it was capped again but with a camera inside. After allowing the system to come to rest for 30 minutes the cap was removed in one movement at 08 seconds and the resulting rush of fissure water can be observed with several pieces of biofilm seen passing the camera at high speed. This simulated the sudden opening of rock due to a seismic event detailing the behavior of fissure water and biofilm under those conditions. The experiment was carried out after the borehole had remained closed for six weeks to eliminate all biofilm in the borehole tube itself. The biofilm pieces that subsequently pass by must therefore come from deeper inside the fissure. The resolution was 640 x 480 at 25 fps and recorded as an AVI file. The file was converted to a MOV format (H264) and deinterlaced using WinX video converter without any other changes. For scale, the diameter of the borehole is 1.5 cm.

**SVIDEO 2 Snapshot 1** at 00:09s. Arrow indicates white strands of biofilm passing by.


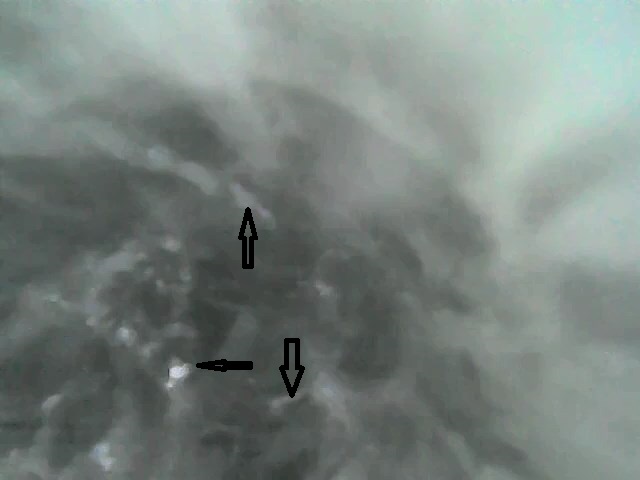


**SVideo 3** Finsch seismic event simulation. At Finsch diamond mine a horizontal borehole of unknown length was sealed with a HD camera inside. After letting the pressure build up for 1 hour the cap was removed in one movement and the effect of sudden fissure water flow was filmed. The cap was removed at 05 seconds. An unexpected side effect of the sudden drop in pressure is the exolution of gas from the fissure water causing additional release of biofilm as is evident at 25 seconds and beyond. The resolution was 640 x 480 at 25 fps and recorded as an AVI file. The file was converted to a MOV format (H264) and deinterlaced using WinX video Converter without any other changes. For scale, the diameter of the borehole is 7.4 cm.

**SVIDEO 3 Snapshot 1** at 00:29s. Black arrows point to exoluted gas bubbles, white arrows point to dislodged biofilm pieces.


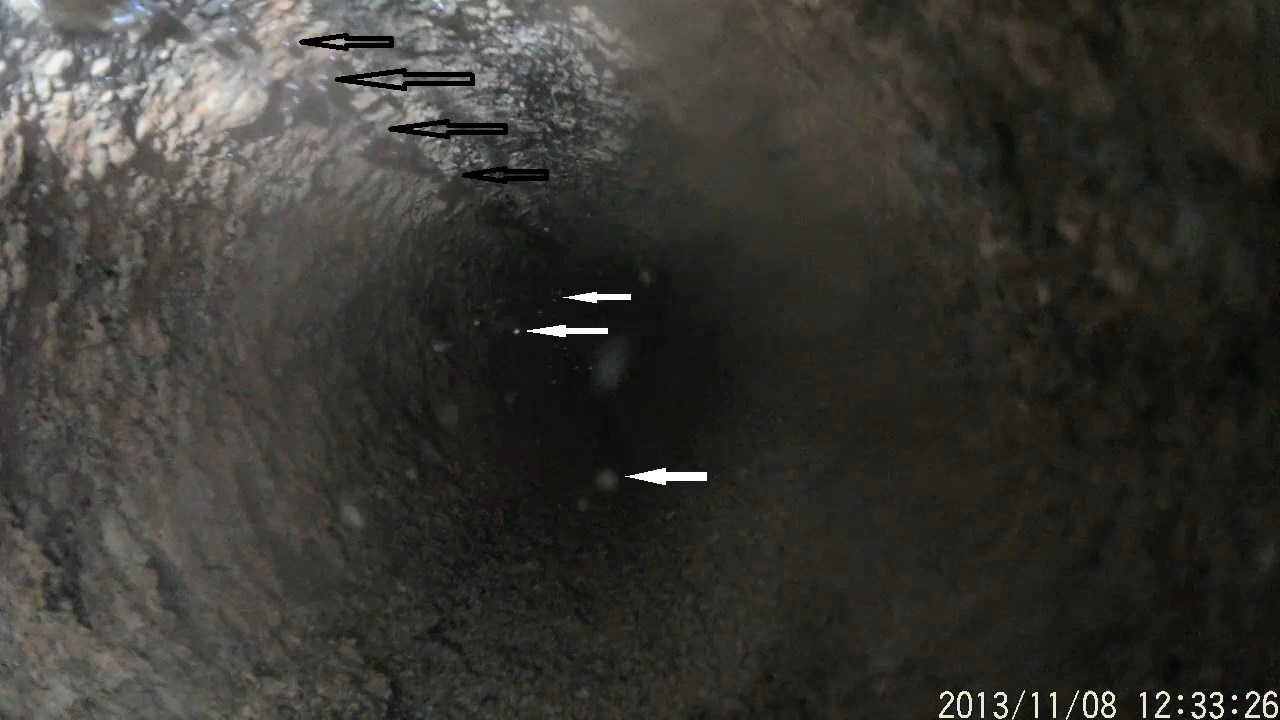


**SVIDEO 4 (WEEK1).** This footage shows the biofilm in a borehole at Star Diamond at a depth of -640 meters. The biofilm growth on the rock face is substantial. The resolution was 640 x 480 at 25 fps and recorded as an AVI file. The file was converted to a MOV format (H264) and deinterlaced using WinX video Converter without any other changes. For scale, the diameter of the borehole is 1.5 cm.

**SVIDEO 4 Snapshot 1** at 00:02s. White arrows point to biofilm.


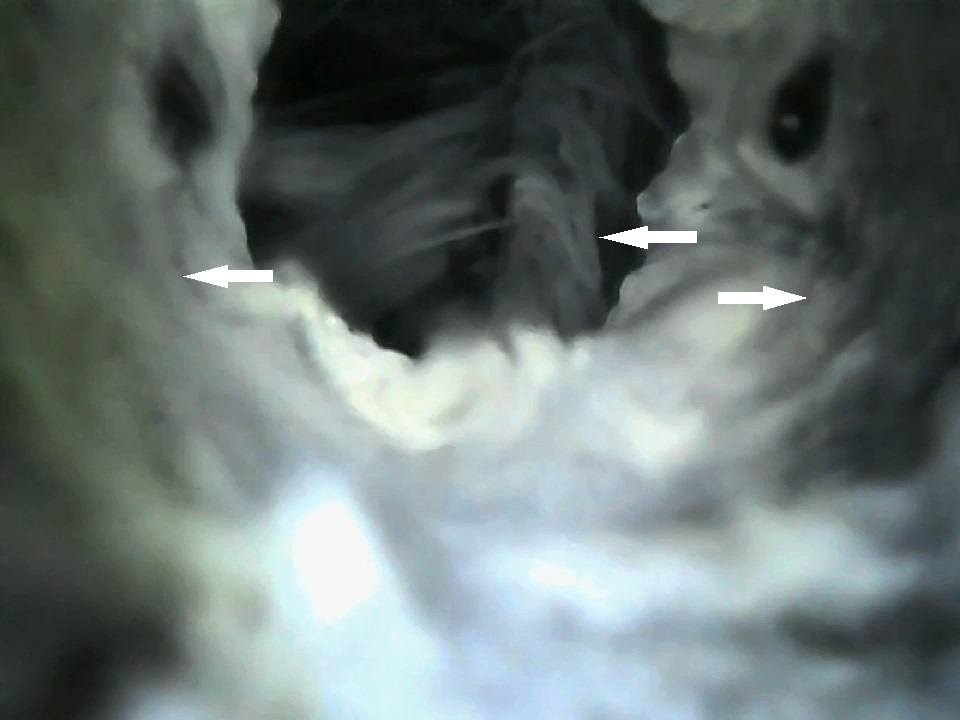


**SVIDEO 5 (Week 6).** This footage shows the absence of biofilm in a borehole at Star Diamond at a depth of -640 meters. This footage is taken from the same perspective of the previous video but after the borehole was capped for 6 weeks creating a cul de sac resulting in the blocking of the fissure water flow. The majority of biofilm has disappeared. The resolution was 640 x 480 at 25 fps and recorded as an AVI file. The file was converted to a MOV format (H264) and deinterlaced using WinX video converter without any other changes. For scale, the diameter of the borehole is 1.5 cm.

**SVIDEO 5 Snapshot 1** at 00:05s. White arrows point to barren rock face inside the borehole.


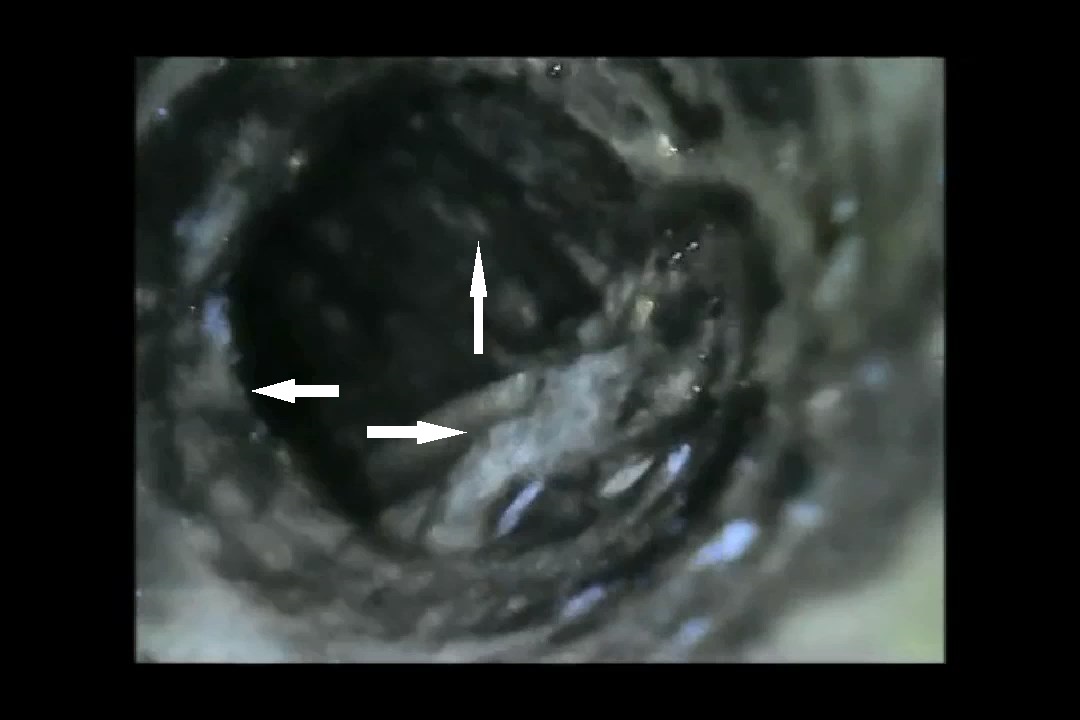


**SVIDEO 6 (WEEK 12).** This footage shows the biofilm in a borehole at Star Diamond at a depth of -640 meters. The footage is taken from the same perspective as SVideo 4 & 5 but after the borehole was uncapped again for 6 weeks and the fissure was allowed to flow unobstructed. Biofilm growth has been established again and is comparable to the growth in SVideo 4. The resolution was 640 x 480 at 25 fps and recorded as an AVI file. The file was converted to a MOV format (H264) and deinterlaced using WinX video converter without any other changes. For scale, the diameter of the borehole is 1.5 cm.

**SVIDEO 6 Snapshot 1** at 00:00s White arrows point to biofilm.


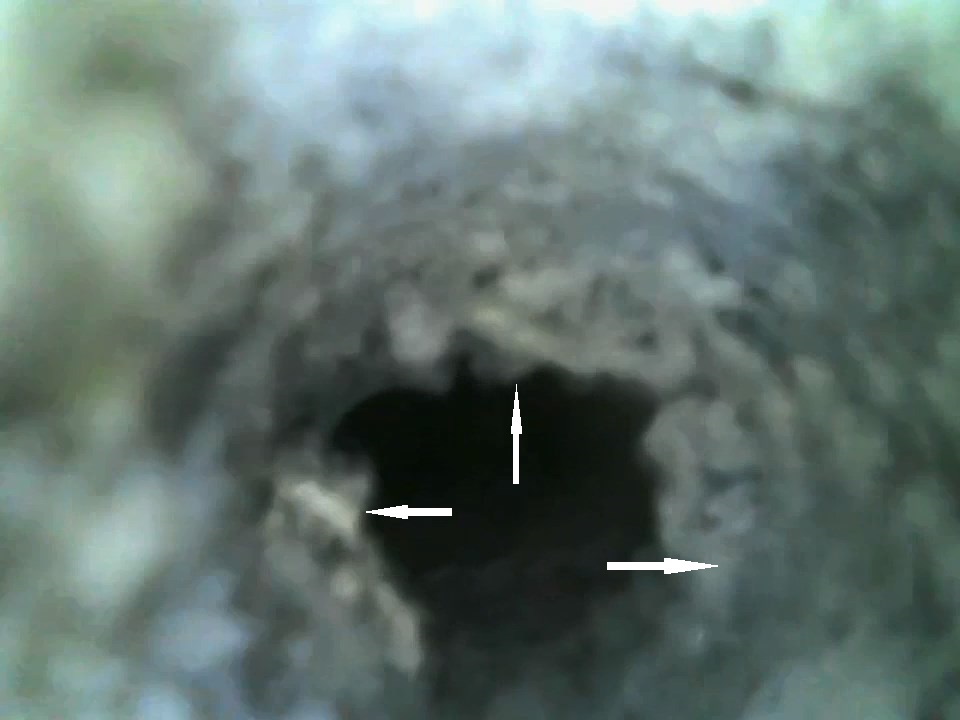


**SVIDEO 6 Snapshot 2** at 00:17s Arrows point to biofilm, asterix indicates the rock face.


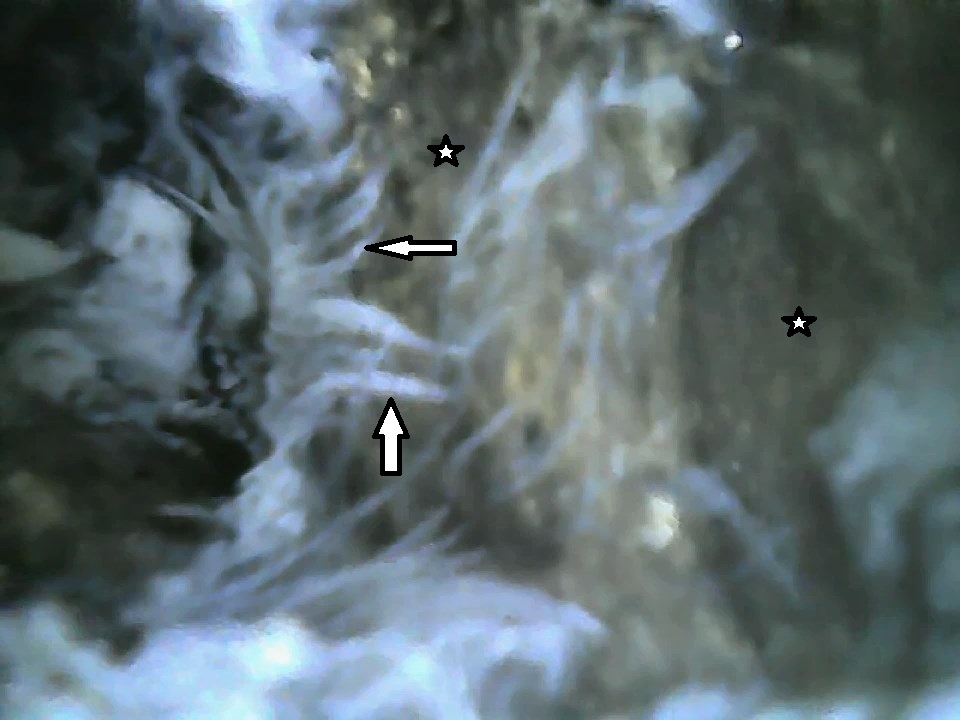


**SVideo 7 Evander Coleoptera capture.** In 2007 at Evander Platinum mine a beetle, subsequently identified as *Hydroglyphus* probably *pusillus* was captured while filling a bottle at a fissure for chemical analysis at a depth of -1.7 km. It was transferred in fissure water with a small piece of biofilm in a 50 ml Falcon tube for observation. The video was made using a Canon MV5i camera. For scale the diameter of the Falcon tube at its widest point is 3 cm.
